# Facet-engineered anatase TiO_2_ nanocrystals with co-exposed {112}, {100}, and {101} facets for enhanced photocatalysis

**DOI:** 10.1039/d6na00419a

**Published:** 2026-09-01

**Authors:** Hiranmay Barma, Pranay Chandra Mandal, Ashis Sarkar, Shakil Ahammad Chowdhury, Ningma Dorzi Sherpa, Chayan Krishna Roy, Swarnendu Roy, Asamanjoy Bhunia, Debabrata Pradhan, Nitish Roy

**Affiliations:** a Department of Chemistry, University of North Bengal Darjeeling W.B. 734013 India nitishroy@nbu.ac.in; b Department of Botany, University of North Bengal Darjeeling W.B. 734013 India; c Materials Science Centre, Indian Institute of Technology Kharagpur W.B. 721302 India; d Department of Chemistry, Inorganic Chemistry Section, Jadavpur University Kolkata W.B. 700032 India

## Abstract

Synthesis of anatase TiO_2_ nanocrystals (NCs) exposing high-index facets remains challenging. Herein, anatase TiO_2_ NCs co-exposing high and low index facets were synthesized by varying the volume ratio of TBAH and DMF. Among the synthesized samples, TiO_2_-30, prepared using 30 mL TBAH and 30 mL DMF, exhibited the highest proportion of rod-like NCs exposing {112}, {100}, and {101} facets. In contrast, TiO_2_-20, synthesized using 20 mL TBAH and 40 mL DMF, predominantly exhibited cube-like NCs exposing {001} and {100} facets. TiO_2_-40, prepared using 40 mL TBAH and 20 mL DMF, retained a rod-like morphology but contained a higher proportion of spherical NCs exposing {101} facets, while TiO_2_-50, synthesized using 50 mL TBAH and 10 mL DMF, primarily consisted of spherical NCs. The photocatalytic activities of the synthesized samples were evaluated using methyl orange (MO) degradation, nitro blue tetrazolium (NBT) reduction, and photocatalytic conversion of toluene to benzaldehyde. Among all the samples, TiO_2_-30 exhibited the highest photocatalytic activity, with rate constants of 11.3 × 10^−2^ min^−1^ for MO degradation and 12.7 × 10^−3^ min^−1^ for NBT reduction, together with a benzaldehyde production rate of 9.92 mg h^−1^ g^−1^. These values are 4.52-, 2.54-, and 1.70-fold higher, respectively, than those obtained using commercial P25 (Degussa). Structural and photoelectrochemical analyses revealed that TiO_2_-30 possesses the highest crystallinity, a lower point of zero charge (ZPC), a more negative flat-band potential, and suppressed charge-carrier recombination compared with the other samples. These characteristics collectively contribute to the superior photocatalytic performance of TiO_2_-30.

## Abstract 2

Enhanced photoexcited charge separation between the {112}, {100}, and {101} facets of anatase TiO_2_ nanocrystals.

## Graphical abstract

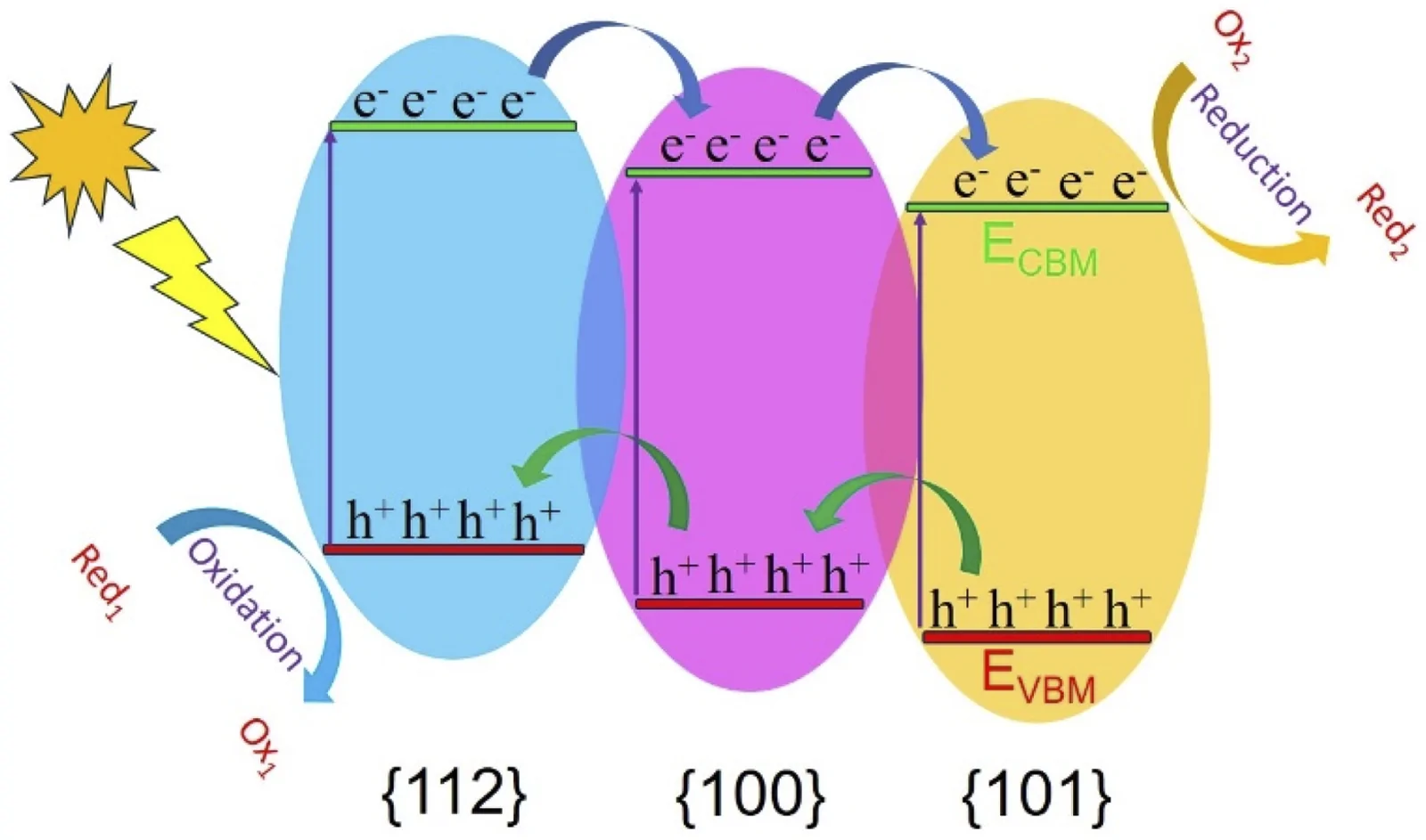

## Introduction

Engineering exposed crystal facets of semiconductor nanocrystals has emerged as an important strategy for improving photocatalytic performance because different facets possess distinct atomic arrangements, coordination environments, and electronic structures.^1–6^ These differences strongly influence adsorption behaviour, charge separation, and surface reaction kinetics. Among various semiconductor photocatalysts, anatase TiO_2_ has been extensively investigated owing to its chemical stability, low cost, and strong oxidative capability. Previous studies have mainly focused on low-index facets such as {101}, {001}, and {100}.^7–14^ In particular, the thermodynamically stable {101} facet generally favours electron accumulation, whereas the higher-energy {001} and {100} facets exhibit enhanced surface reactivity due to the presence of under-coordinated surface atoms.^10–14^

Recently, co-exposure of multiple facets has attracted significant interest because facet-dependent charge separation can create surface heterojunctions within a single crystal, thereby suppressing electron–hole recombination and enhancing photocatalytic activity.^14^ One of the most extensively investigated strategies in anatase TiO_2_ photocatalysis is facet engineering to expose high-energy surfaces, particularly the {001} and {100} facets, owing to their relatively high surface energies.^7–9,15^ {001} and {100} facets of anatase TiO_2_ are low-index but high in surface energy due to the presence of five coordinate Ti (Ti_5C_) and two coordinate O (O_2C_) at the surfaces (Fig. 1).^15^ The surface unsaturation along with the dangling bonds of the {001} and {100} facets results in their high surface energies of 0.90 J m^−2^ and 0.53 J m^−2^, respectively.^15–17^ On the other hand, low-energy {101} facets contain 50% Ti_5C_ and 50% Ti_6C_ at the surface with zigzag geometry, resulting in a lower surface energy (0.43 J m^−2^).^15,17^ It has been established that the high-energy facets of anatase TiO_2_ exhibit enhanced surface reactivity such as adsorption of small molecules.^18–20^ However, anatase TiO_2_ that exposes only high-energy facets such as thin sheets with nearly 100% {001} facets might not be suitable for photocatalytic reactions due to higher e^−^/h^+^ pair recombination.^10^ However, the co-existence of both high-energy and low-energy facets in a nanocrystal (NC) has been suggested to result in higher e^−^/h^+^ pair separation and enhanced photocatalytic activity.^11,12,14,21^ The enhanced charge separation in an anatase TiO_2_ NC between the high-energy {001}/{100} and low-energy {101} facets is ascribed to the presence of a surface heterojunction between these facets. However, high-index facets, *e.g.*, {112} facets of anatase TiO_2_, have high surface energy (0.65–0.73 J m^−2^) due to their higher surface unsaturation and unique surface chemistry.^22,23^ {112} facets of anatase TiO_2_ possess 100% Ti_5C_ and 100% O_2C_.^15,16,24–27^ These result in higher surface energy of the {112} facets. The surface geometry and coordination numbers of (001), (101), (100) and {112} facets of anatase TiO_2_ are displayed in Fig. 1.

**Fig. 1:**
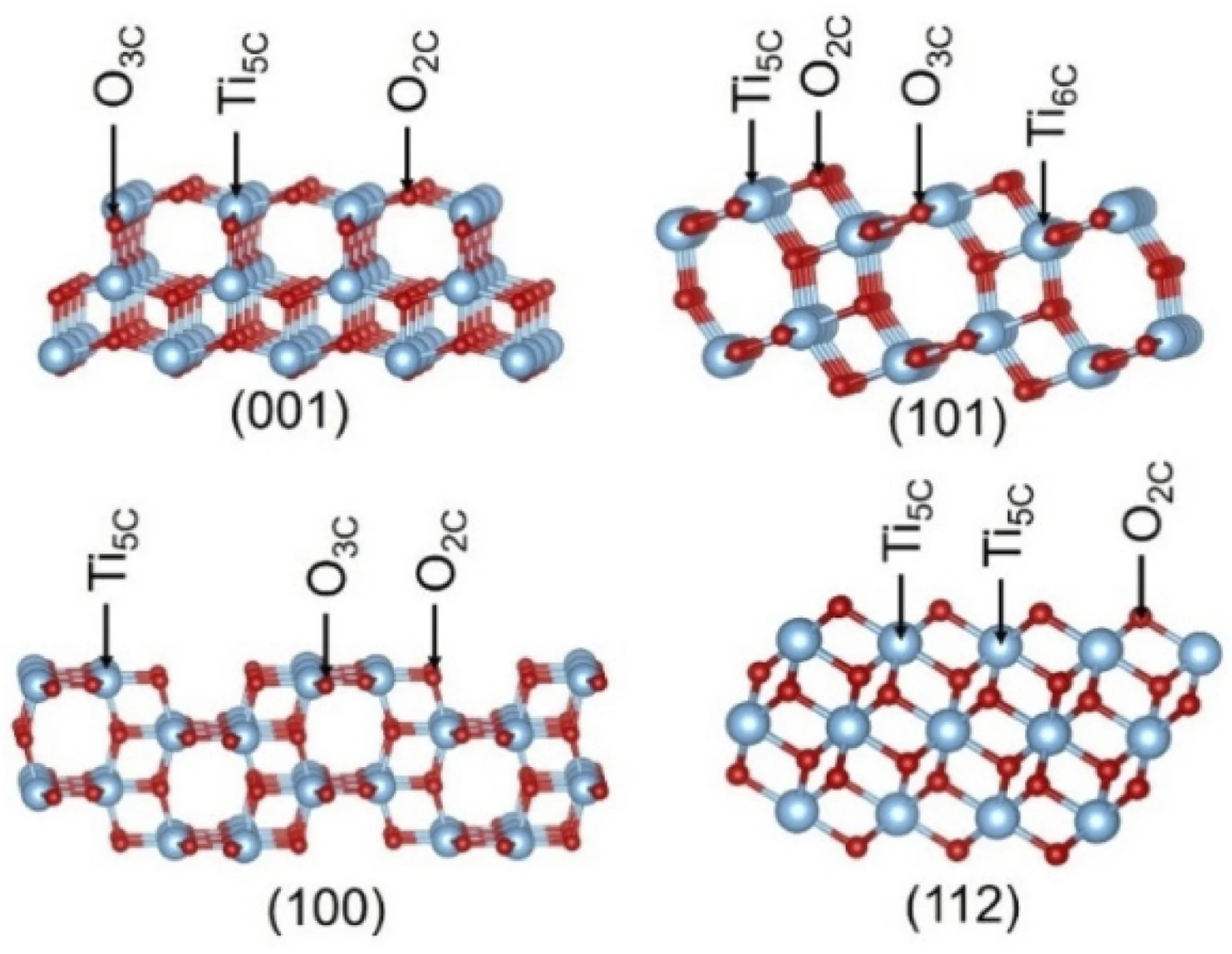
Ball and stick models of anatase TiO_2_ surfaces exposing (001), (101), (100) and (112) planes, showing different coordination numbers.

Theoretical calculations have been performed to elucidate the relationship between the surface energy and chemical reactivity of anatase TiO_2_ facets. Mino *et al.* systematically investigated CO adsorption on four TiO_2_ crystallographic surfaces—(101), (100), (001), and (112)—using two hybrid DFT functionals, PBE0 and B3LYP.^28^ Their results revealed that at 50% surface coverage (*θ* = 0.5), the (112) surface consistently exhibits the strongest CO binding energy among the examined facets, irrespective of the computational method. Specifically, under the PBE0 functional, the (112) surface showed the highest adsorption energy of 34.2 kJ mol^−1^, while under the B3LYP functional, it maintained the strongest interaction with a binding energy of 23.1 kJ mol^−1^.

Complementary experimental and theoretical studies by Joutsuka *et al.* further confirmed the high reactivity of the (112) facet.^29^ They reported that the (112) surface exhibits the strongest water adsorption energy (−1.01 eV), compared to the (101) (−0.80 eV) and (100) (−0.63 eV) surfaces, and also facilitates efficient charge separation. Nevertheless, to the best of our knowledge, a systematic experimental investigation correlating the adsorption characteristics of TiO_2_ facets with their photocatalytic performance toward different probe reactions, such as toluene oxidation, methyl orange (MO) degradation, and nitro blue tetrazolium (NBT) reduction, remains limited. Therefore, in the present work, we systematically investigate the adsorption and photocatalytic properties of anatase TiO_2_ exposing both high-index {112} and low-index {100} and {101} facets.

Anatase TiO_2_ nanocrystals were synthesized hydrothermally by varying the DMF/TBAH ratio while maintaining a constant total solvent volume. Detailed synthesis procedures and characterization methods are provided in the SI. FESEM and TEM analysis revealed systematic morphology evolution with increasing TBAH concentration (Fig. S1 and S2). The size of the NCs in the sample TiO_2_-20 was found to be in the range of 35 to 55 nm. The magnified FESEM image [inset of Fig. S2(a)] and TEM image [Fig. S1(a)] reveal that the shape of the TiO_2_ NCs is primarily cubic. With an increase in the volume of TBAH to 30 mL (DMF volume 30 mL) during the synthesis of TiO_2_ NCs (denoted as TiO_2_-30), the size was found to increase, ranging from 30 to 60 nm [Fig. S1(b) and S2(b)] with the morphology primarily consisting of rod-like shapes. TiO_2_-20 predominantly exhibits cube-like nanocrystals (71%), whereas TiO_2_-30 is dominated by rod-like nanocrystals (61%). With a further increase in the TBAH volume to 40 mL (DMF volume 20 mL) during the synthesis of TiO_2_ NCs (TiO_2_-40), the size was found to increase (40 to 90 nm), while the morphology is a mixture of rod-like (51%) and spherical shapes. TiO_2_-50 mainly consists of spherical nanocrystals (73%) (Table S1 and Fig. S3). This systematic evolution in particle morphology, together with the quantitative shape distribution, demonstrates that the DMF/TBAH ratio plays an important role in directing the preferential growth and morphological development of TiO_2_ nanocrystals during hydrothermal crystallization.

To comprehensively evaluate the influence of crystal facet engineering on photocatalytic performance, two representative liquid-phase photocatalytic reactions were selected. Methyl orange (MO) degradation was employed as a model reaction for assessing the oxidative degradation capability of TiO_2_ toward organic pollutants, whereas the selective liquid-phase oxidation of toluene to benzaldehyde was chosen as a model organic transformation requiring efficient charge separation and oxygen activation. Investigating both reactions under liquid-phase conditions enables a comprehensive assessment of how the coexistence of different exposed crystal facets influences photocatalytic oxidation activity, oxygen reduction reaction selectivity, and charge-carrier dynamics.

Powder XRD and Raman spectroscopy, as shown in Fig. S6, confirm the formation of phase-pure anatase TiO_2_ without detectable secondary phases. All characteristic Raman-active anatase modes are clearly observed. The XRD patterns reveal increasing peak intensity and peak sharpening with increasing TBAH concentration, indicating improved crystallinity. TiO_2_-30 exhibits relatively high crystallinity with moderate crystallite size, suggesting an optimized balance between crystal growth and structural order. Detailed calculations of crystallite size and crystallinity are shown from Tables S3 to S6.

The exposed crystal facets were investigated using HRTEM and HAADF-STEM analyses (Fig. S7 and 2). The TiO_2_-30 sample exhibits well-resolved lattice fringes corresponding to the (112), (−112), and (200) planes, as shown in Fig. 2(b). Sides of the rods are zigzag-shaped and parallel to (112) planes, indicating the sides are exposed with {112} facets. The FFT pattern was indexed to the (112), (−112), and (200) reflections (Fig. 1(c)). The interplanar distances and angles between these planes matched exactly with the crystallographic data along the [0−12] zone axis, as shown in Fig. S4 and S5.^30,31^ Furthermore, these experimental results were directly compared with the crystallographic model of anatase TiO_2_ projected along the [0−12] zone axis, where the angular relationships between the (112), (−112), and (200) planes are reproduced.^32–34^ The ball-and-stick model therefore serves as the crystallographic reference supporting the experimental indexing [Fig. 2(a–c)]. The measured interplanar spacings and angular relationships agree well with the crystallographic geometry of anatase TiO_2_, confirming the presence of co-exposed {112}, {100}, and {101} facets. HRTEM analysis, as shown in Fig. S7, indicates that the elongated rod-like morphology appears to favour the intermediate-energy {112} facets together with low-energy {100}/{101} facets. Based on the crystallographic data and the average length-to-width dimensions obtained from the TEM analysis (Fig. S1–S3), the elongated rod-like nanocrystals in the sample TiO_2_-30 are estimated to expose approximately 1% {001}, 49% {100}, 33% {112}, and 16% {101} facets. In comparison, the cube-like nanocrystals are estimated to expose approximately 33.33% {100} and 66.66% {001} facets.

**Fig. 2:**
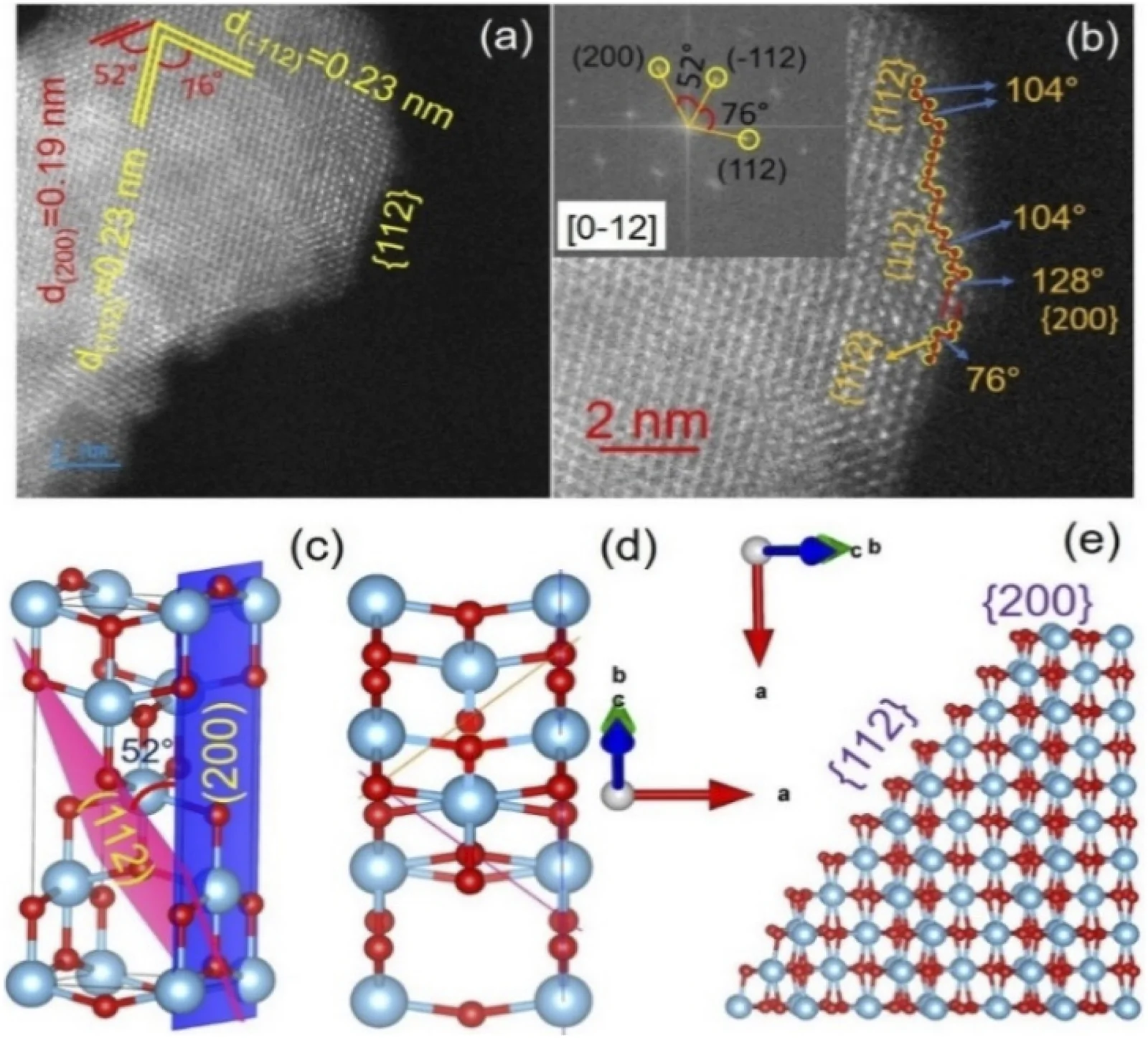
(a) HRTEM image of the sample TiO_2_-30, (b) HAADF-STEM image of a representative TiO_2_-30 nanocrystal (selected portion of sloping side faces of the elongated rod-like bipyramidal NCs) showing the measured interplanar spacings and angles between crystallographic planes, and (c) magnified HAADF-STEM image from the upper region of the nanocrystal highlighting the exposed facets and corresponding angular relationships; inset shows the FFT pattern indexed to the (112), (−112), and (200) planes. (d) Anatase TiO_2_ unit cell projected along [0−12] showing (112), (−112), and (200) planes are parallel. (e) Surface projections of {112} and {200} facets along [0−12].

The optical characteristics of all samples were examined using ultraviolet-visible (UV-vis) spectroscopy [Fig. 3(a)]. The anatase TiO_2_ samples exhibit an electronic absorption threshold of around 400 nm. The band gaps of the TiO_2_ samples were determined by applying the Kubelka–Munk rule, as shown in Fig. 3(b). The band gaps of TiO_2_-20, TiO_2_-30, TiO_2_-40, and TiO_2_-50 were calculated to be 3.52, 3.42, 3.35, and 3.22 eV, respectively. The higher bandgap of the sample TiO_2_-20 indicates its lower size, as revealed by TEM, Raman and XRD analysis. Electrochemical Mott–Schottky (M–S) experiments are crucial to measure the flat-band potential and donor density as well as to understand the capacitance behaviour at the catalyst surface–electrolyte interface. The M–S experiments were performed at 1000 Hz in 0.5 M Na_2_SO_4_ using Ag/AgCl saturated in KCl as a reference electrode, a Pt mesh as a counter electrode and TiO_2_ coated on a glassy carbon disc electrode as a working electrode in a conventional three electrode system. The typical plots of 1/*C*^2^*vs.* potential (V *vs.* NHE) are shown in Fig. 3(c). The intercepts of the plot on the *X*-axis provide the flat band potential, thereby revealing their relative CB positions. The sample TiO_2_-30 showed the highest negative flat band potential of −0.31 V *vs.* RHE, followed by TiO_2_-40 (−0.21 V *vs.* RHE), TiO_2_-50 (−0.17 V *vs.* RHE) and TiO_2_-20 (−0.05 V *vs.* RHE), respectively. The higher negative flat-band potential of TiO_2_-30 (−0.31 V *vs.* RHE) could be due to the higher density of elongated rod-like NCs exposing {112} facets and the higher crystallinity of the samples as revealed by XRD (Fig. S6(b) and Tables S3–S6).

**Fig. 3:**
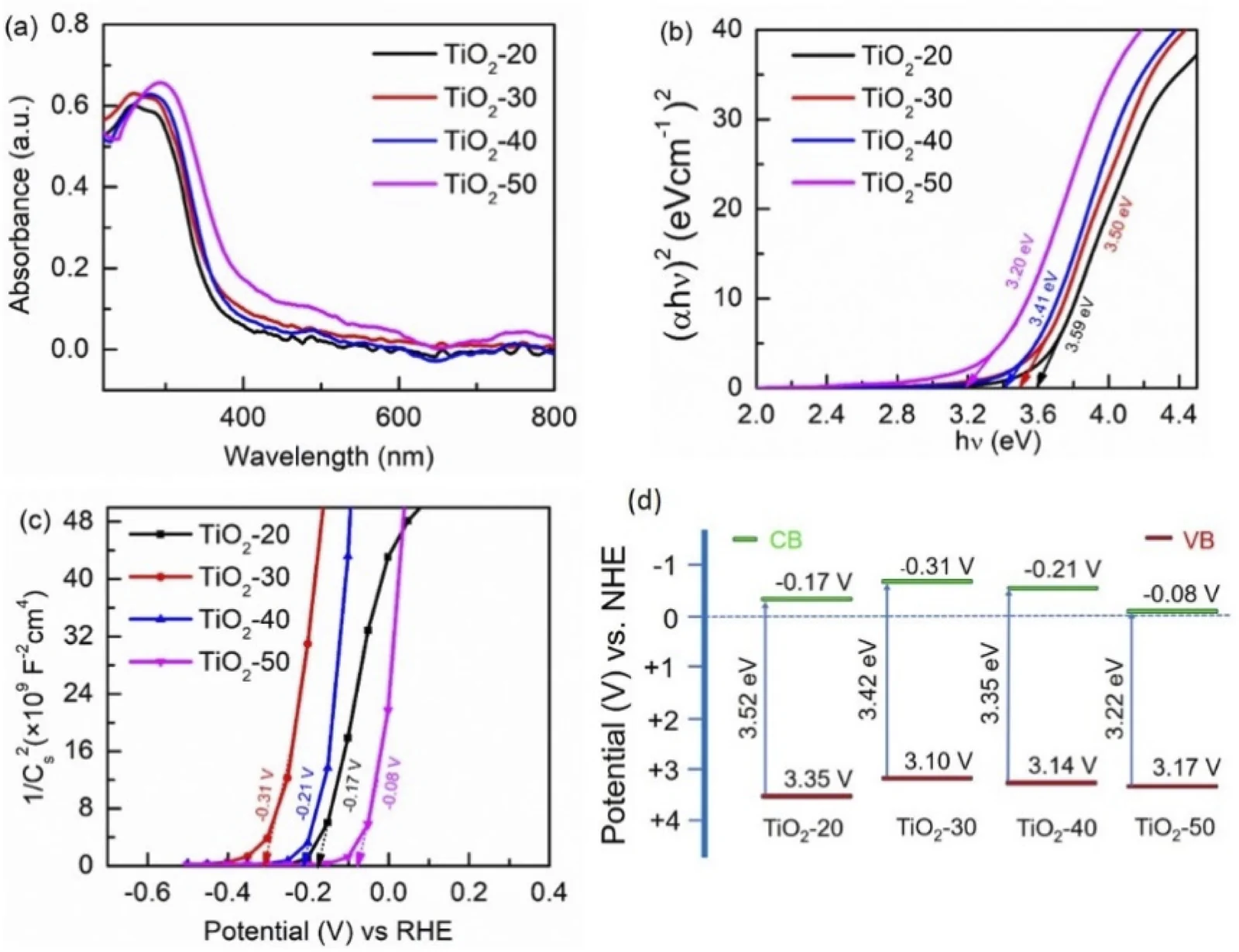
(a) UV-vis diffuse reflectance spectra of the as-synthesized TiO_2_ samples. (b) Corresponding optical band-gap plots derived from the Kubelka–Munk function. (c) Mott–Schottky plots of different TiO_2_ samples measured at 1000 Hz in 0.5 M Na_2_SO_4_ electrolyte. (d) Schematic representation of the conduction band (CB) and valence band (VB) positions of the synthesized TiO_2_ samples.

The photoluminescence (PL) spectra can reveal information on carrier capture, migration, conversion, and separation. It has also been used for evaluating the separation of photogenerated e^−^/h^+^ pairs.^35^ The PL peaks reveal the recombination of photogenerated e^−^/h^+^ pairs, while the intensity indicates their extent of recombination. Higher photocatalytic performance is associated with a lower PL intensity.^36^ However, low PL does not always mean better photoresponse, especially when most of the energy is lost through non-radiative processes. PL only shows the radiative recombination of electrons and holes. However, TiO_2_ nanocrystals can also lose charge carriers through non-radiative recombination.^37–39^Fig. 4(a) displays the PL spectra of the samples, with the emission intensity being highest for TiO_2_-50 and lowest for TiO_2_-30. The TiO_2_-30 sample has the highest percentage of elongated rod-like bipyramidal NCs, which expose high-energy {112} and {100} facets along with the low-energy {101} facets. The presence of both high-energy and low-energy facets in anatase TiO_2_ is known to reduce the e^−^/h^+^ pair recombination.^40–43^ Therefore, the lowest e^−^/h^+^ pair recombination of the sample TiO_2_-30 as revealed by PL analysis is due to the presence of both high-energy and low-energy facets. PL analysis indicates that the extent of e^−^/h^+^ pair recombination for different samples would be in the order TiO_2_-30 < TiO_2_-40 < TiO_2_-20 < TiO_2_-50. Furthermore, the photogenerated charge carrier separation and transfer rate were estimated using photocurrent responses, as shown in Fig. 4(b). All TiO_2_ samples exhibit clear and reproducible photocurrent responses under light on/off cycles, confirming good photoresponsive reversibility. Samples TiO_2_-20 and TiO_2_-50 show similar types of photoresponses during light on/off cycles with steady-state photocurrent. However, their photocurrent densities were lower. The highest photocurrent density was observed for the sample TiO_2_-30 followed by the sample TiO_2_-40. The photoresponse decay profiles of TiO_2_-30 and TiO_2_-40 were similar, suggesting the presence of trap states in these samples. The presence of trap states could originate from the fact that these samples have high-energy {112} exposed facets. During the crystal growth and shape evolution of the high-energy {112} exposed facets, some defects might be introduced into the crystal structures. However, their photocurrent densities were higher, clearly showing the surface heterojunction between the {112}, {100} and {101} facets enhances charge separation.^44,45^ Surface charge is a significant factor during the adsorption process for cationic or anionic species. ZPC measurement provides the pH value where the net surface charge is neutral. Therefore, ZPC provides information regarding the surface nature of the solids. ZPC was measured using 20 mg of catalyst in 10 mL of DI water in 0.01 M NaCl.^46,47^ The pH of each solution was adjusted within the range of 4 to 12 by adding 0.1 M HCl or 0.1 M NaOH. The mixtures were then shaken for 2 h using a shaker and allowed to equilibrate for 24 h. After equilibration, the final pH of each solution was measured, and the change in pH was plotted against the initial pH values, as shown in Fig. 4(c). The ZPC values of the different samples were found to be in the order TiO_2_-30 (6.28) < TiO_2_-40 (7.02) < TiO_2_-20 (7.48) < TiO_2_-50 (7.78), indicating that the surface of TiO_2_-30 is positively charged compared to other samples.

**Fig. 4:**
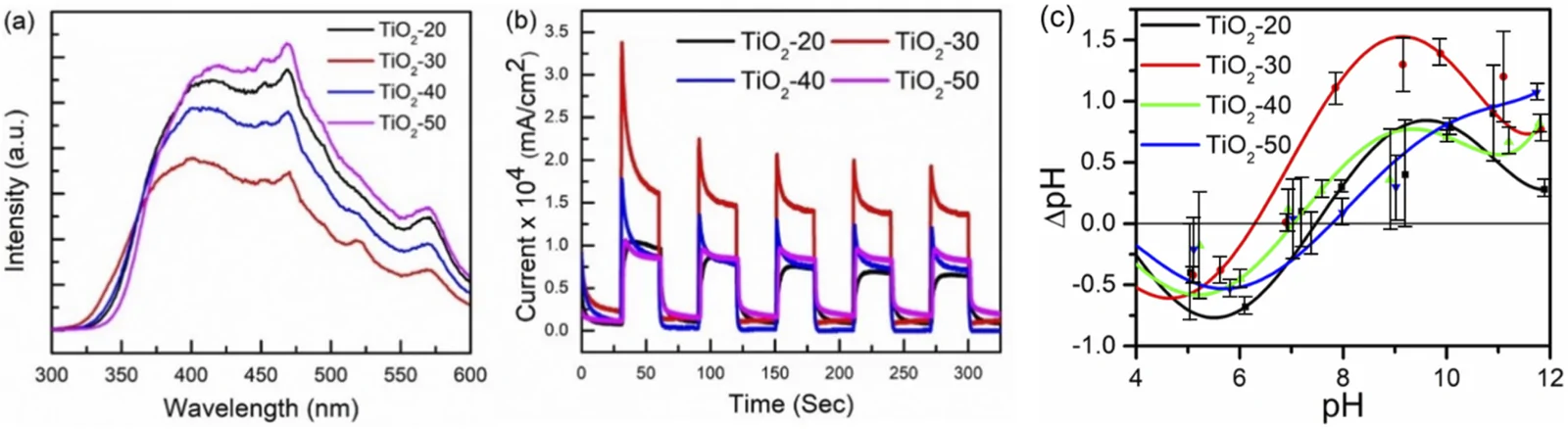
(a) Photoluminescence (PL) spectra of the as-synthesized TiO_2_ samples. (b) Transient photocurrent responses of different TiO_2_ samples under periodic light on/off irradiation. (c) Point of zero charge (ZPC) plots of different TiO_2_ samples measured in 0.01 M NaCl solution.

The photocatalytic performance of the TiO_2_ nanocrystals was evaluated using photocatalytic oxidation of toluene, nitroblue tetrazolium (NBT) reduction, and methyl orange (MO) degradation under UV irradiation (Fig. 5 and S8–S11). Among all synthesized samples, TiO_2_-30 exhibits the highest photocatalytic activity. The benzaldehyde production rate during photocatalytic toluene oxidation reaches 9.92 mg h^−1^ g^−1^ for TiO_2_-30, which is significantly higher than those of other TiO_2_ samples and commercial P25 (Table 1). The photocatalytic oxidation predominantly yields benzaldehyde with high selectivity, while only minor benzyl alcohol formation is observed. Control experiments [Fig. 5(a and b)] using radical scavengers indicate that superoxide radicals 
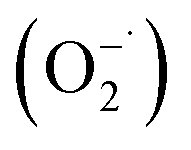
 play a dominant role in benzaldehyde formation. Addition of benzoquinone significantly suppresses product formation, confirming the crucial involvement of superoxide radicals. Electron scavenging experiments also reveal the importance of photogenerated electrons in the photocatalytic pathway. These results suggest that reduction of molecular oxygen to 
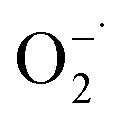
 is a key step governing selective oxidation of toluene.

**Fig. 5:**
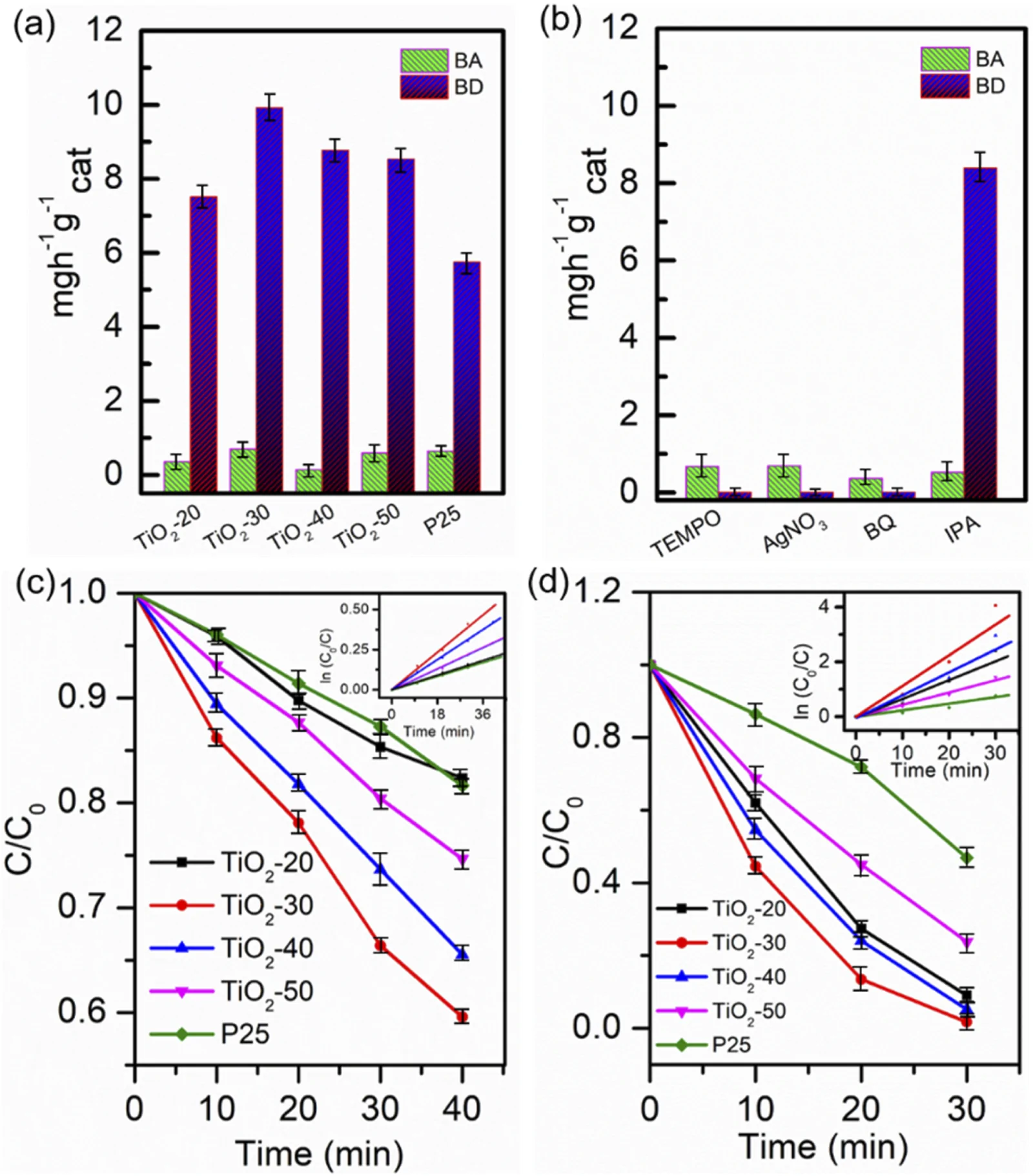
(a) Photocatalytic oxidation of toluene to benzaldehyde (BD) and benzoic acid (BA) over different anatase TiO_2_ photocatalysts under UV irradiation. (b) Scavenger-controlled photocatalytic oxidation of toluene using TiO_2_-30 in the presence of different radical scavengers. (c) Photocatalytic reduction of nitroblue tetrazolium (NBT) by generated 
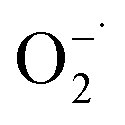
 radicals in the presence of different TiO_2_ photocatalysts under 360 nm UV irradiation; inset shows the corresponding pseudo-first-order kinetic plots [ln(*C*_0_/*C*) *versus* irradiation time]. (d) Photocatalytic degradation of methyl orange (MO) under 360 nm UV irradiation using different TiO_2_ photocatalysts; inset shows the corresponding pseudo-first-order kinetic plots [ln(*C*_0_/*C*) *versus* irradiation time].

**Table 1:** BD and BA production rate, rate constant for NBT reduction (*k*_NBT_) and rate constant of MO degradation (*k*_MO_) by the different photocatalysts

Catalyst	BD (mg h^−1^ g^−1^)	BA (mg h^−1^ g^−1^)	BD selectivity	*k*_NBT_ (min^−1^)	*k*_MO_ (min^−1^)
TiO_2_-20	7.51	0.35	95.54	5.1 × 10^−3^	6.8 × 10^−2^
**TiO**_**2**_ **-30**	**9.92**	**0.70**	**93.40**	**12.7 × 10^−^** ^**3**^	**11.3 × 10^−^** ^**2**^
TiO_2_-40	8.77	0.14	98.42	10.4 × 10^−3^	8.5 × 10^−2^
TiO_2_-50	8.53	0.59	93.53	7.3 × 10^−3^	4.5 × 10^−2^
P25	5.75	0.65	89.84	5.0 × 10^−3^	2.5 × 10^−2^

NBT degradation experiments further confirm enhanced superoxide radical generation over TiO_2_-30 (Fig. S10 and S11). Similarly, TiO_2_-30 exhibits the fastest MO degradation kinetics and achieves complete degradation within 30 min, whereas commercial P25 requires substantially longer irradiation time under identical conditions (Fig. S7). The enhanced photocatalytic activity of TiO_2_-30 can be attributed to the presence of {112} facets and its higher crystallinity, which could enhance adsorption properties along with enhanced charge separation.

The superior performance of TiO_2_-30 is proposed to arise from facet-dependent charge separation between co-exposed {112}, {100}, and {101} facets. The coexistence of multiple facets may generate internal electric fields and surface heterojunctions that promote directional migration of photogenerated electrons and holes, thereby suppressing recombination and enhancing surface redox reactions. In particular, the high-index {112} facets are expected to provide a higher density of active surface sites and facilitate oxidation processes, whereas the lower-energy {100}/{101} facets may favour reduction reactions. The cooperative interaction between these facets therefore plays a crucial role in improving photocatalytic efficiency.

The presence of under-coordinated Ti_5C_ sites at the TiO_2_ surface is expected to facilitate the formation of surface Ti–OH species, which can participate in photocatalytic oxidation reactions.^29^ The observed lower production rate of BA compared to BD suggests that multiple reactive pathways are involved in toluene oxidation. Upon photoexcitation, holes (h^+^) can either generate hydroxyl radicals (˙OH) or directly oxidize toluene to form benzyl radicals. In addition, reactive oxygen species (ROS), particularly superoxide radicals 
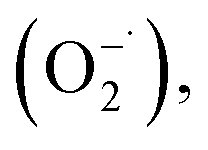
 can also contribute to benzyl radical formation. Once formed, the benzyl radical can follow two competing pathways: reaction with ˙OH to form BA or reaction with 
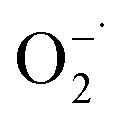
 to yield BD. The predominance of BD formation indicates that the pathway involving 
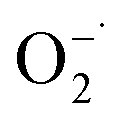
 is more favourable under the present reaction conditions. To elucidate the role of different reactive species, controlled scavenger experiments were performed [Fig. 5(b)]. In the presence of TEMPO, a significant suppression of product formation was observed, confirming the involvement of radical intermediates such as e^−^, h^+^, 
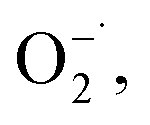
 hydroperoxyl radicals (HOO˙), and ˙OH in the reaction. Further insights were obtained using specific scavengers: AgNO_3_ (electron scavenger), benzoquinone (BQ, 
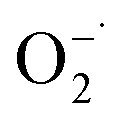
 scavenger), and isopropanol (IPA, hole scavenger). The addition of AgNO_3_ led to a marked decrease in product formation, highlighting the crucial role of photogenerated electrons. Similarly, the use of BQ significantly suppressed BD formation, indicating that 
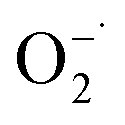
 radicals play a dominant role in the oxidation pathway leading to BD. In contrast, the relatively smaller effect of hole scavenging suggests that h^+^ and ˙OH are less involved in determining the selectivity toward BD, although they may contribute to benzyl radical formation. These results suggest that the reduction of O_2_ by photogenerated e^−^ to form 
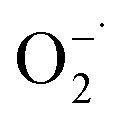
 is a key step in the reaction pathway and may act as a rate-limiting process. Overall, the photocatalytic oxidation of toluene over TiO_2_ NCs appears to be governed by a superoxide-mediated pathway, leading predominantly to benzaldehyde formation (Fig. 6).

**Fig. 6:**
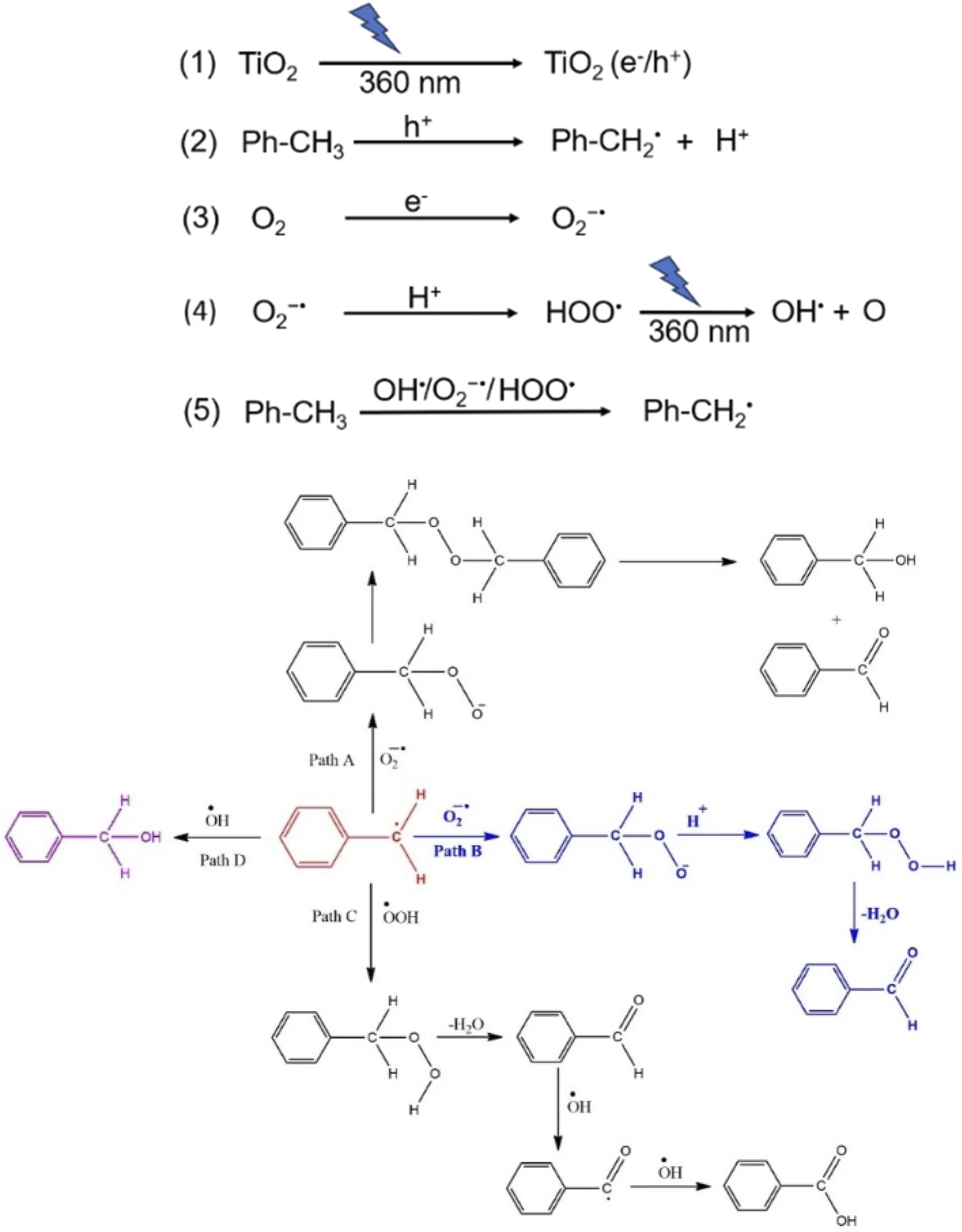
Plausible photocatalytic toluene oxidation in the presence of reactive species. Left panel: photoexcitation of TiO_2_ and the reactions involving photoexcited electrons and holes. Right panel: rearrangement of these reactive species for the formation of different products.

In this work, anatase TiO_2_ nanocrystals were synthesized by varying the DMF/TBAH ratio. The optimized TiO_2_-30 sample exhibits an elongated rod-like morphology with co-exposed {112}, {100}, and {101} facets. Owing to the presence of a higher proportion of high-index {112} facets in conjunction with the {100} and {101} facets, TiO_2_-30 demonstrated superior photocatalytic activity toward oxidative degradation of MO and toluene conversion to benzaldehyde *via* reductive activation of O_2_ to 
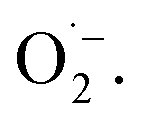
 The higher proportion of {112} facets in TiO_2_-30 is further reflected by its lower ZPC, more negative flat-band potential, and enhanced transient photocurrent response, indicating improved charge separation and stronger surface reactivity. These findings demonstrate that the incorporation of high-index {112} facets, together with an appropriate combination of low-index facets, is an effective strategy for enhancing the photocatalytic performance of anatase TiO_2_ nanocrystals.

## Conflicts of interest

The authors declare no competing financial interests.

## Supplementary Material

NA-008-D6NA00419A-s001

## Data Availability

The data supporting this article have been included as part of the supplementary information (SI). Supplementary information: experimental details, additional FESEM and HRTEM images, adsorption studies, UV-vis spectra, HPLC spectra, photocatalytic control experiments, and supplementary characterization data. See DOI: https://doi.org/10.1039/d6na00419a.
