# Supplementary material for: Facet-engineered anatase TiO2 nanocrystals with co-exposed {112}, {100}, and {101} facets for enhanced photocatalysis
Source: Nanoscale Adv. 2026 Sep 1;8(19):5526–34. doi: 10.1039/d6na00419a (PMC13559540; doi:10.1039/d6na00419a)

Supplementary Information (SI) for Nanoscale Advances.
This journal is © The Royal Society of Chemistry 2026
Supplementary Information (SI) for Nanoscale Advances.
This journal is © The Royal Society of Chemistry

Facet-Engineered Anatase TiO_2 Nanocrystals with Co-Exposed {112}, {100}, and {101} Facets for Enhanced Photocatalysis

Hiranmay Barma,[a] Pranay Chandra Mandal,[a] Ashis Sarkar,[b] Shakil Ahammad Chowdhury,[c] Ningma Dorzi Sherpa,[a] Chayan Krishna Roy,[a] Swarnendu Roy,[b] Asamanjoy Bhunia,[d] Debabrata Pradhan,[c] and Nitish Roy[a,*]

[a] Department of Chemistry, University of North Bengal, Darjeeling, W.B., 734013, India
[b] Department of Botany, University of North Bengal, Darjeeling, W.B., 734013, India
[c] Materials Science Centre, Indian Institute of Technology Kharagpur, W.B., 721302, India
[d] Department of Chemistry, Inorganic Chemistry Section, Jadavpur University, Kolkata, 700032, W.B., India

Experimental section

Chemicals. Titanium isopropoxide (TTIP) extrapure, Tetra-butyl ammonium hydroxide (TBAH) 0.1N aq. solution extrapure AR, Nitro Blue Tetrazolium Chloride (NBT) extrapure AR, 99% were purchased from SRL. N,N-Dimethyl formamide (DMF) anhydrous, 99.8%, 2,2,6,6 tertamethylpiperidin-1-oxyl (TEMPO), Degussa P25, Nafion solution were purchased from Sigma-Aldrich. Toluene, Acetonitrile anhydrous HPLC grade, Benzoquinone, Sodium sulfate anhydrous, Silver nitrate, Methyl orange, Acetone were purchased from Merck India. All the chemicals were used as received.

Synthesis of TiO_2 NCs. A hydrothermal method was employed to synthesize TiO_2 NCs. In a typical synthesis, 1 mL (3 mmole) of TTIP was added to a mixture of TBAH (20 to 50 mL) and DMF (40 to 10 mL) keeping total volume fixed at 60 mL. On addition of TTIP to the mixture of TBAH and DMF, the solution became white turbid and stirred for about 10 minutes in a magnetic stirrer at room temperature. The reaction mixture was then transferred into an autoclave, sealed and heated using a Muffle furnace for 24 hour at 200°C. Following the hydrothermal reaction, the temperature was reduced to room temperature naturally and a white precipitate of TiO_2 NCs was collected through centrifugation, washed several times with distilled water and 2-propanol. The product was then dried in a hot air oven overnight and calcined at 500 °C for 1 hour to remove the impurities present in the sample.

Characterization. JEOL-2010 Field Emission Scanning Electron Microscope (FESEM), SIGMA VP FESEM, ZEISS and a JEM 2100 PLUS High Resolution Transmission Electron Microscope (HRTEM) and FIB-FEG Scanning Electron Microscope AURIGATM Compact were employed for the morphology and microstructure analysis of the as-synthesized TiO_2 samples. Powder X-Ray Diffraction (XRD) with Cu Kα radiation (SmartLab X-RAY DIFFRACTOMETER, Rigaku) and Horiba Labram HR Evo were used for phase analysis. A CHI electrochemical workstation with three electrode configuration was used for electrochemical analysis. UV-Visible Spectrophotometry (Agilent 8453 UV-Vis Spectroscopy System and Hitachi U-2910 Spectrophotometer) and Photon Technology International Inc. ASOC-10 Fluorimeter were used for optical and emission studies, respectively. Photocatalytic toluene oxidation products such as benzaldehyde (BD), and

benzyl alcohol (BA) were characterized by HPLC (Agilent 1290 UHPLC, Santa Clara, USA) using 1:1 acetonitrile/water mixture in a C18 column.

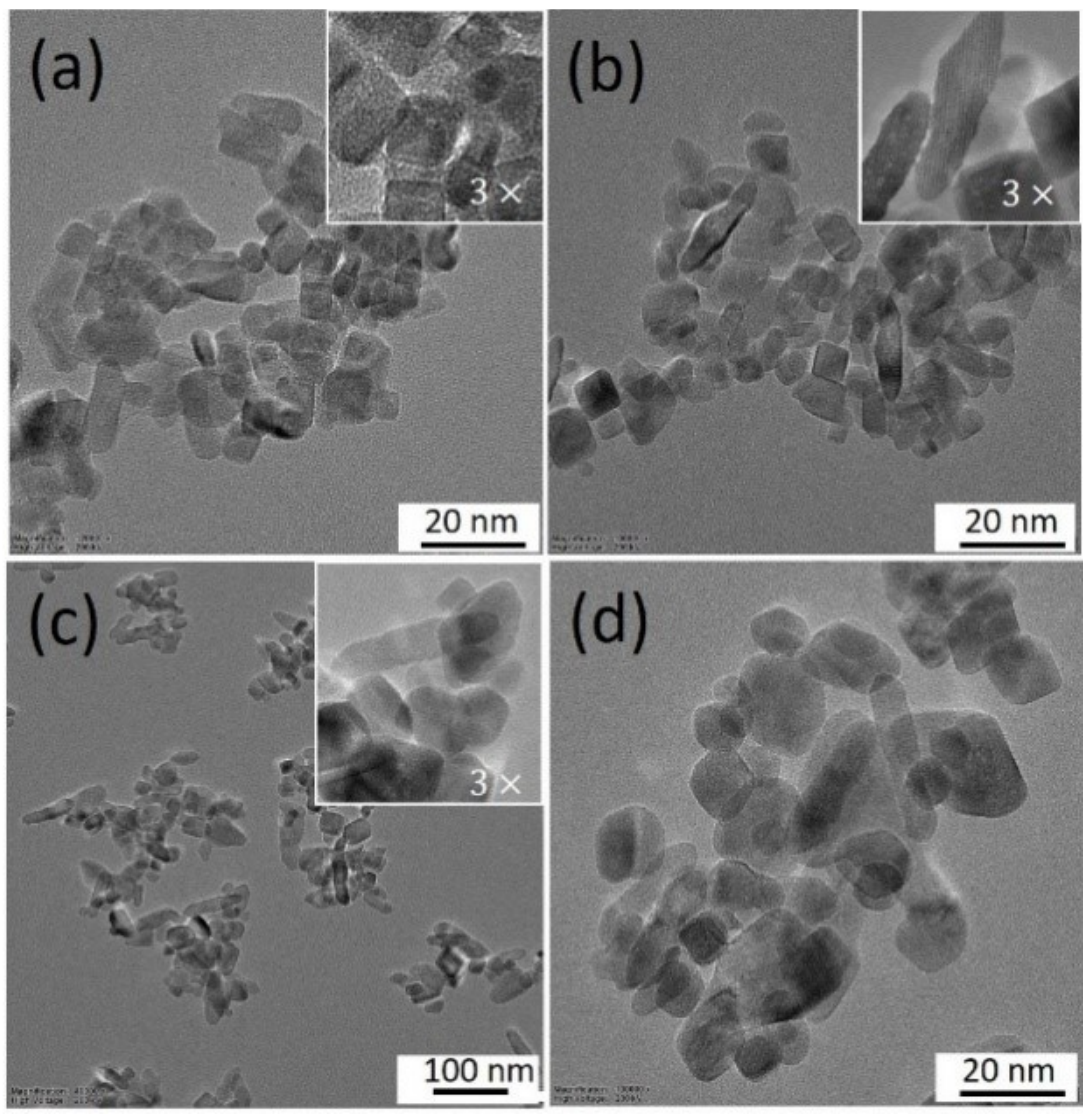

Figure S1. TEM images of the samples (a) TiO_2-20, (b) TiO_2-30, (c) TiO_2-40, (d) TiO_2-50. In sets of Figure (a), (b) and (c) show the corresponding magnified TEM images.

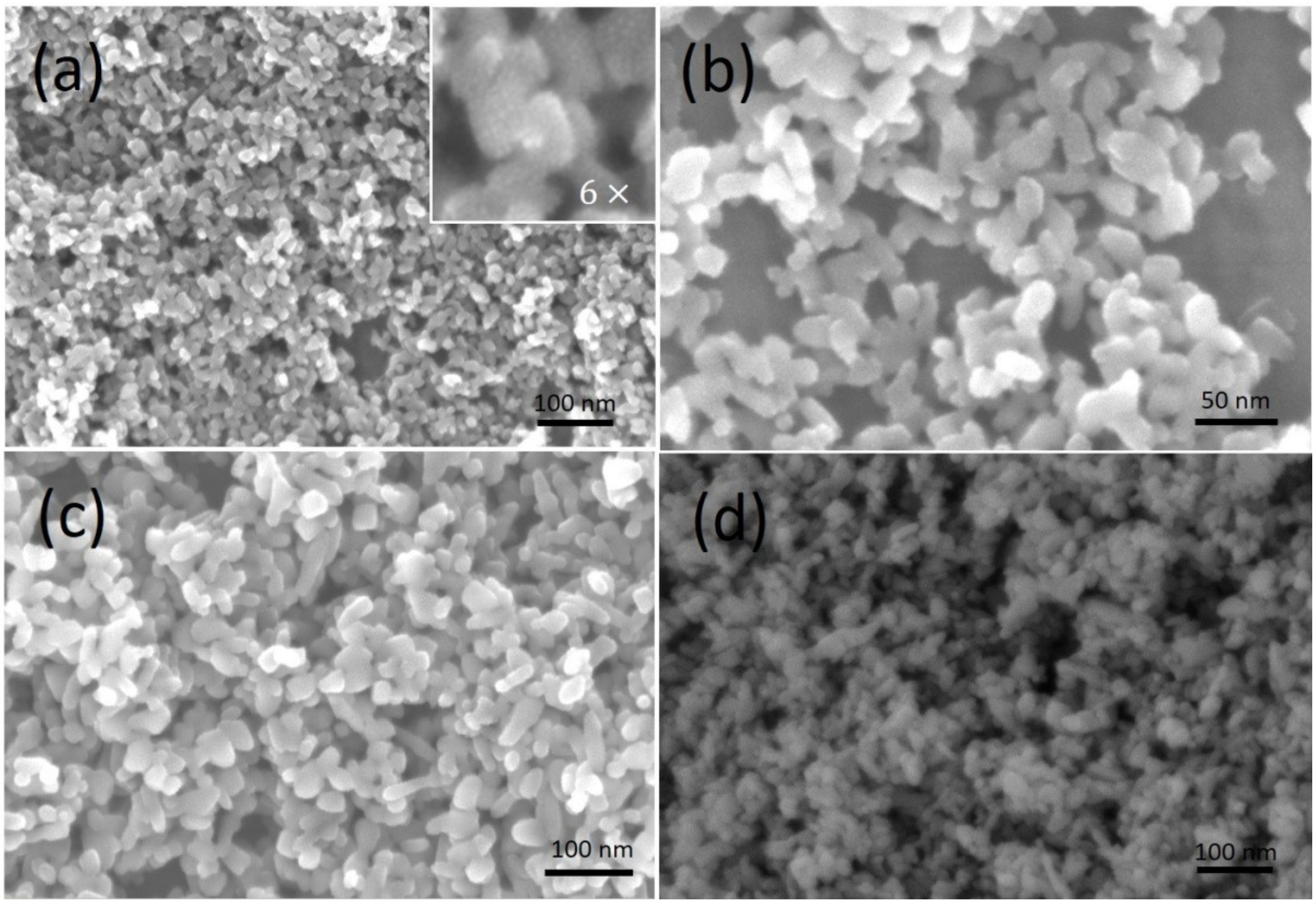

Figure S2. FESEM images of the samples (a) TiO_2-20, (b) TiO_2-30, (c) TiO_2-40, (d) TiO_2-50. In sets of Figure (a) shows the corresponding magnified SEM images.

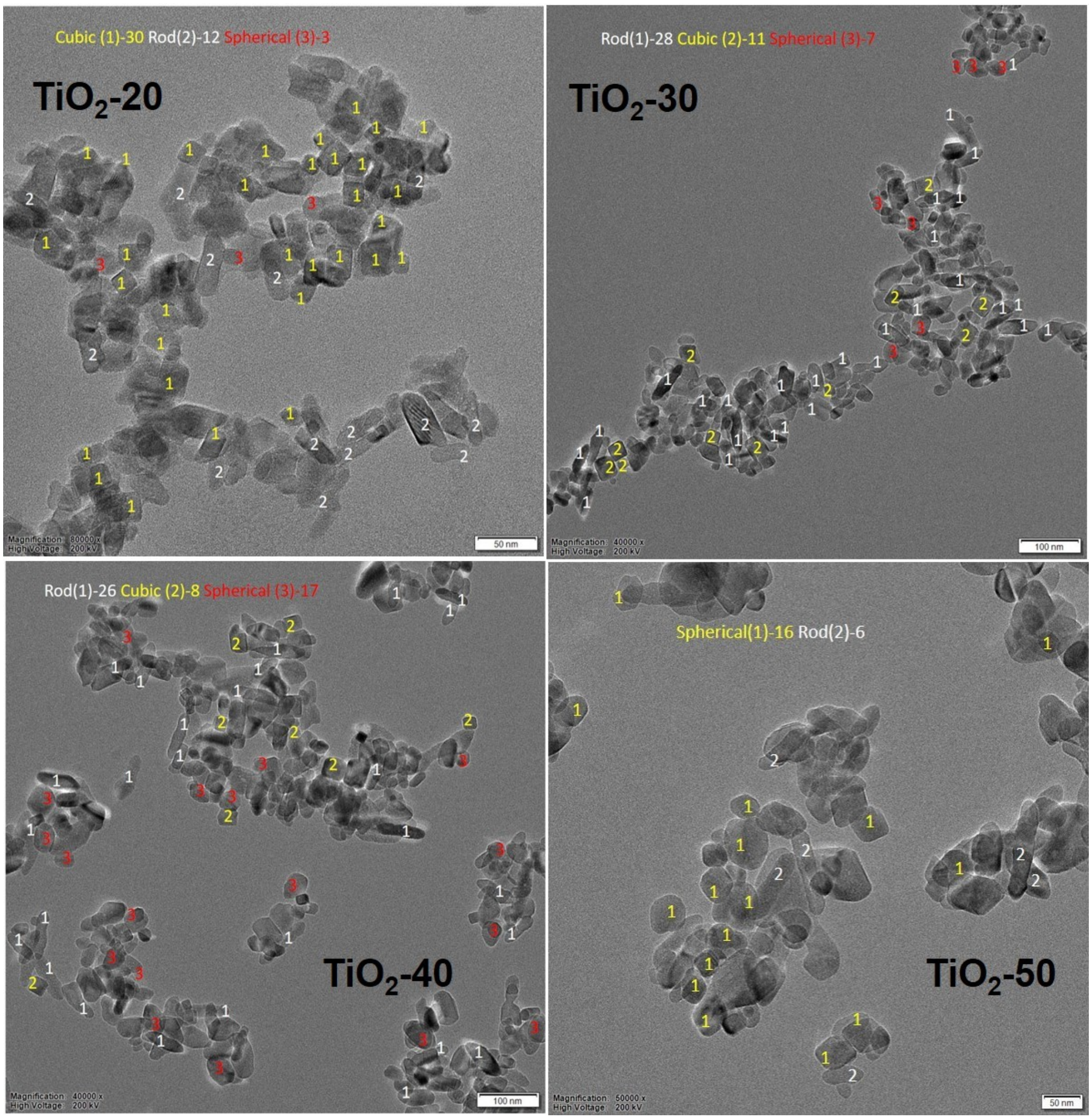

Figure S3. Representative TEM images of TiO_2-20, TiO_2-30, TiO_2-40, and TiO_2-50 showing particle morphology with variation of the DMF/TBAH ratio. The numbers indicate the counted particles corresponding to the respective morphologies, providing a quantitative assessment of the relative distribution of cubic, rod-like, elongated rod-like, and spherical nanocrystals.

Table S1. Quantitative analysis of particle morphology distribution for the synthesized TiO_2 samples.

Synthesized Samples	% of Rod type	% of Cube type	% of Spherical type
TiO_2-20	28	71	2-3
TiO_2-30	61	23	15
TiO_2-40	51	15	33
TiO_2-50	27	3-5	73

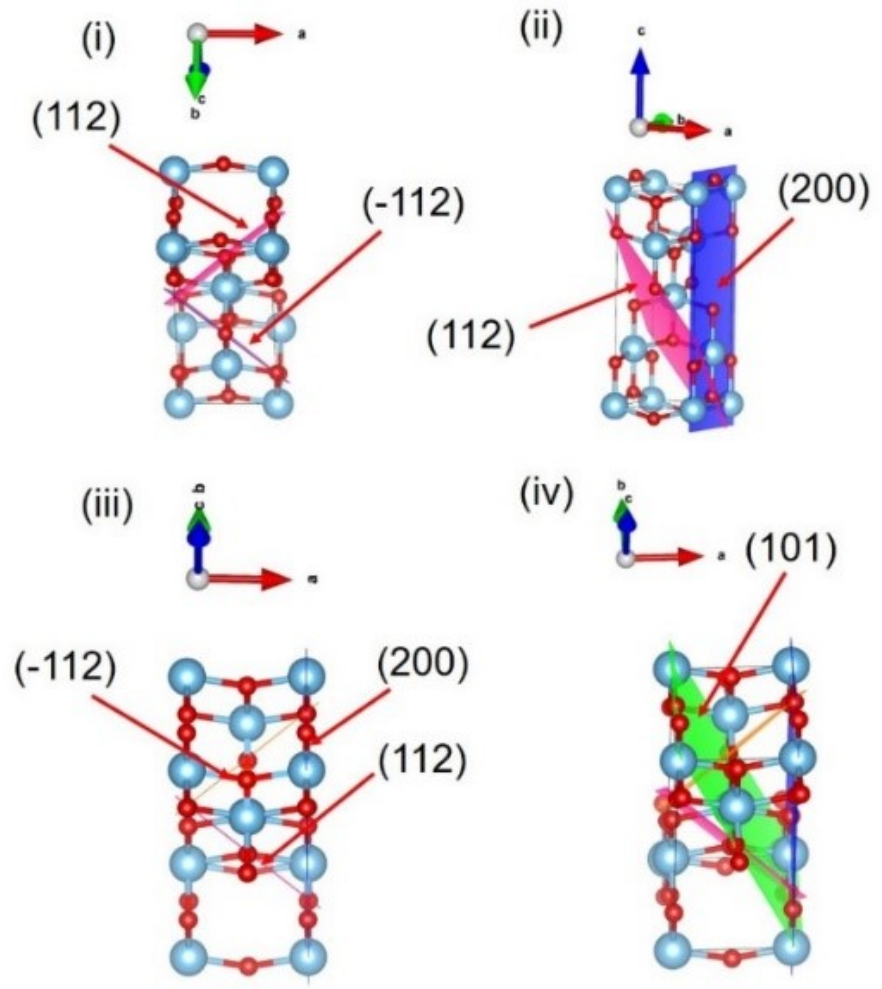

Figure S4. Unit cell of anatase TiO_2 showing (i) (112) and (-112) planes, (ii) (112) and (200) planes, (iii) (112), (-112) and (200) planes along [0-12] direction, and (iv) same as (iii) but with an additional (101) plane showing along [0-12] direction clearly confirming the (101) planes are not parallel to [0-12] direction.

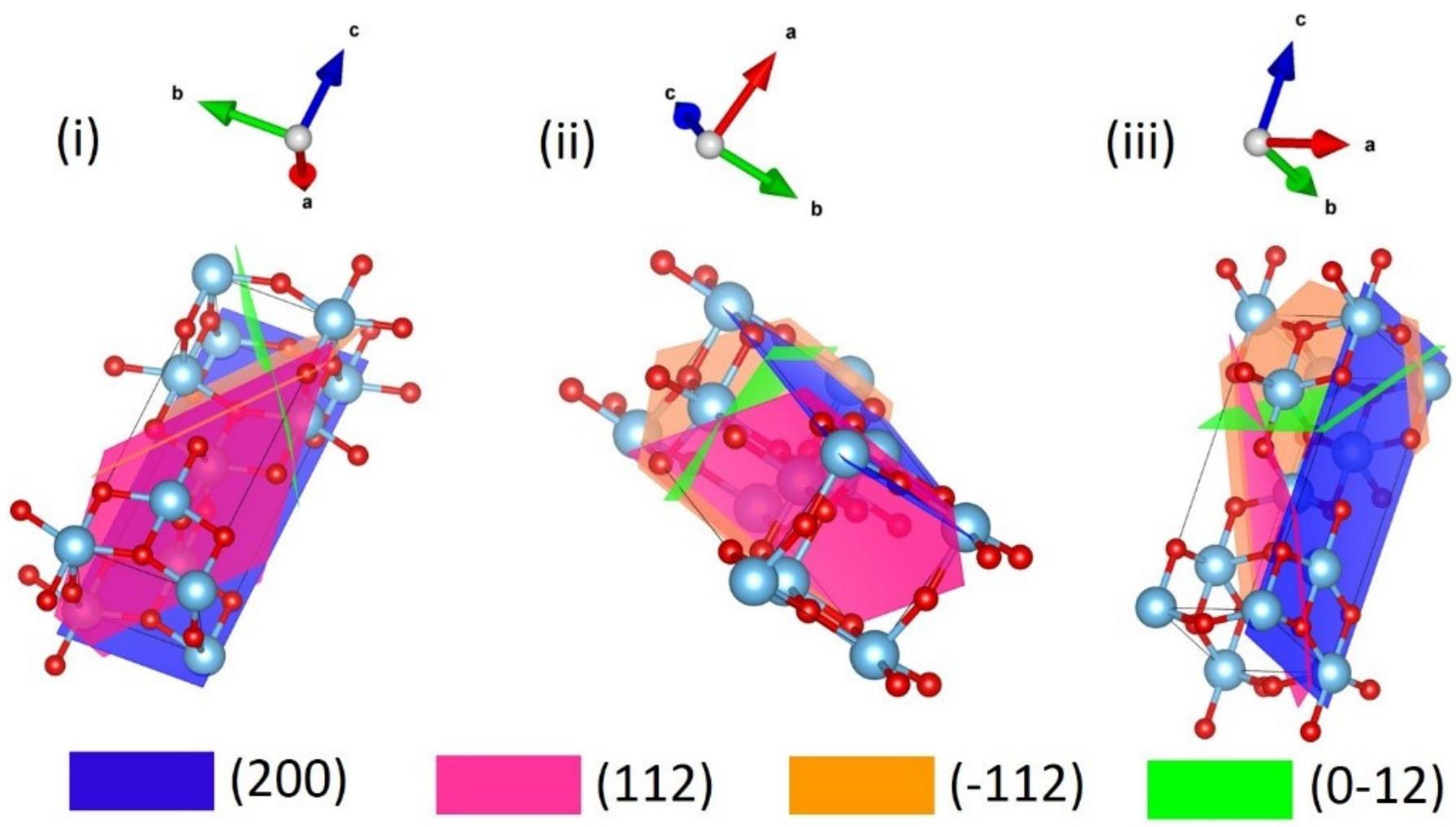

Figure S5. Unit cell of anatase TiO_2 showing (i) (-112) and (0-12) planes are orthogonal to each other, (ii) (200) and (0-12) planes are orthogonal to each other, (iii) (112) and (0-12) planes are orthogonal to each other. (200), (112), (-112) and (0-12) planes are shown by blue, pink, orange and green colour shed, respectively.

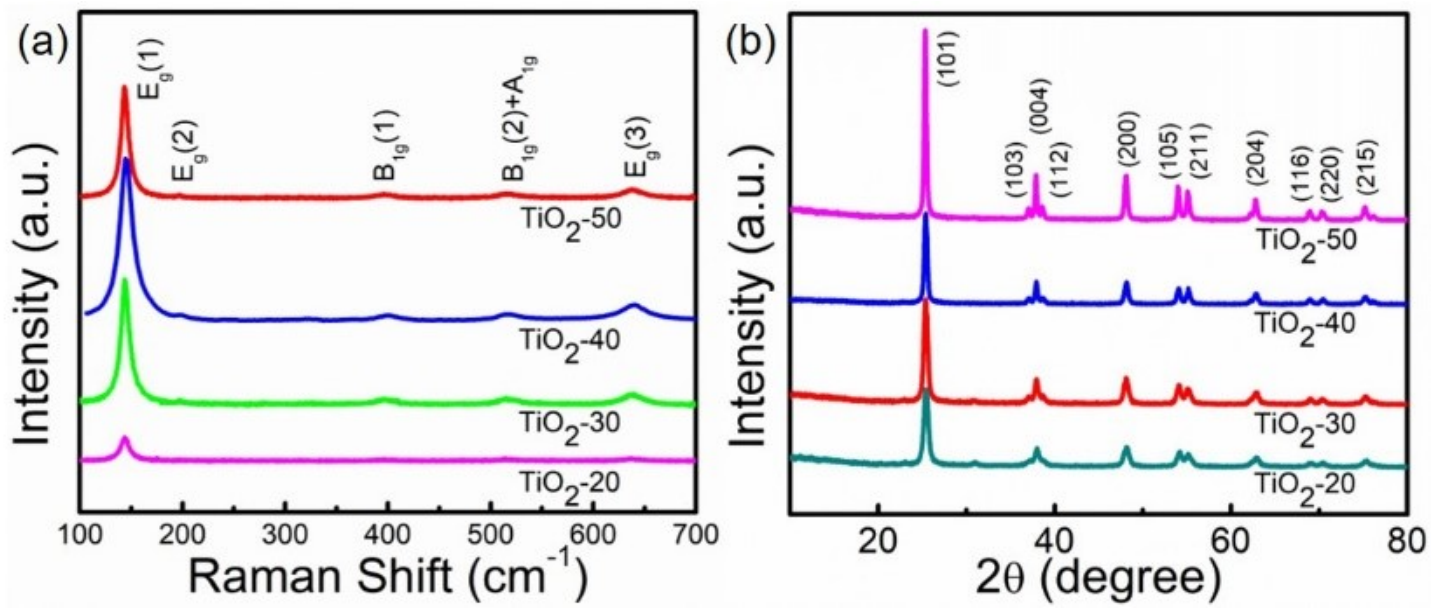

Figure S6. (a) Raman spectra and (b) powder XRD pattern of the as-synthesized TiO_2 samples.

Table S2. Crystallinity, average crystallite size

Samples	Crystallinity (%)	Average size (nm)
TiO_2-20	54.18	11.36
TiO_2-30	62.01	12.59
TiO_2-40	55.18	18.07
TiO_2-50	55.75	26.83

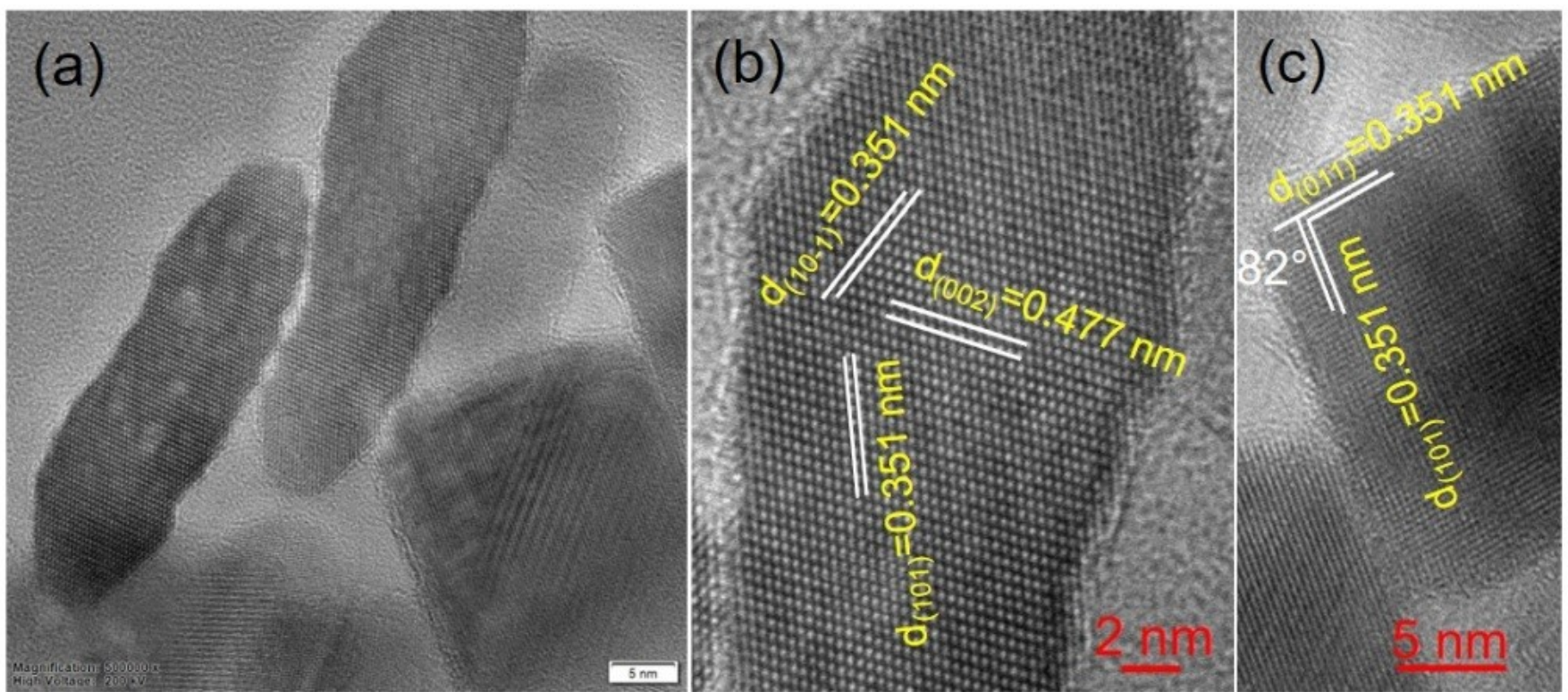

Figure S7. HRTEM image of the sample TiO_2-30 (a) showing different shapes which include cubic, rod-like structures with zigzag sides, (b) magnified image from a NC with elongated rod-like bipyramidal shape showing lattice fringes, and (c) magnified image from a NC with cube-like shape showing lattice fringes.

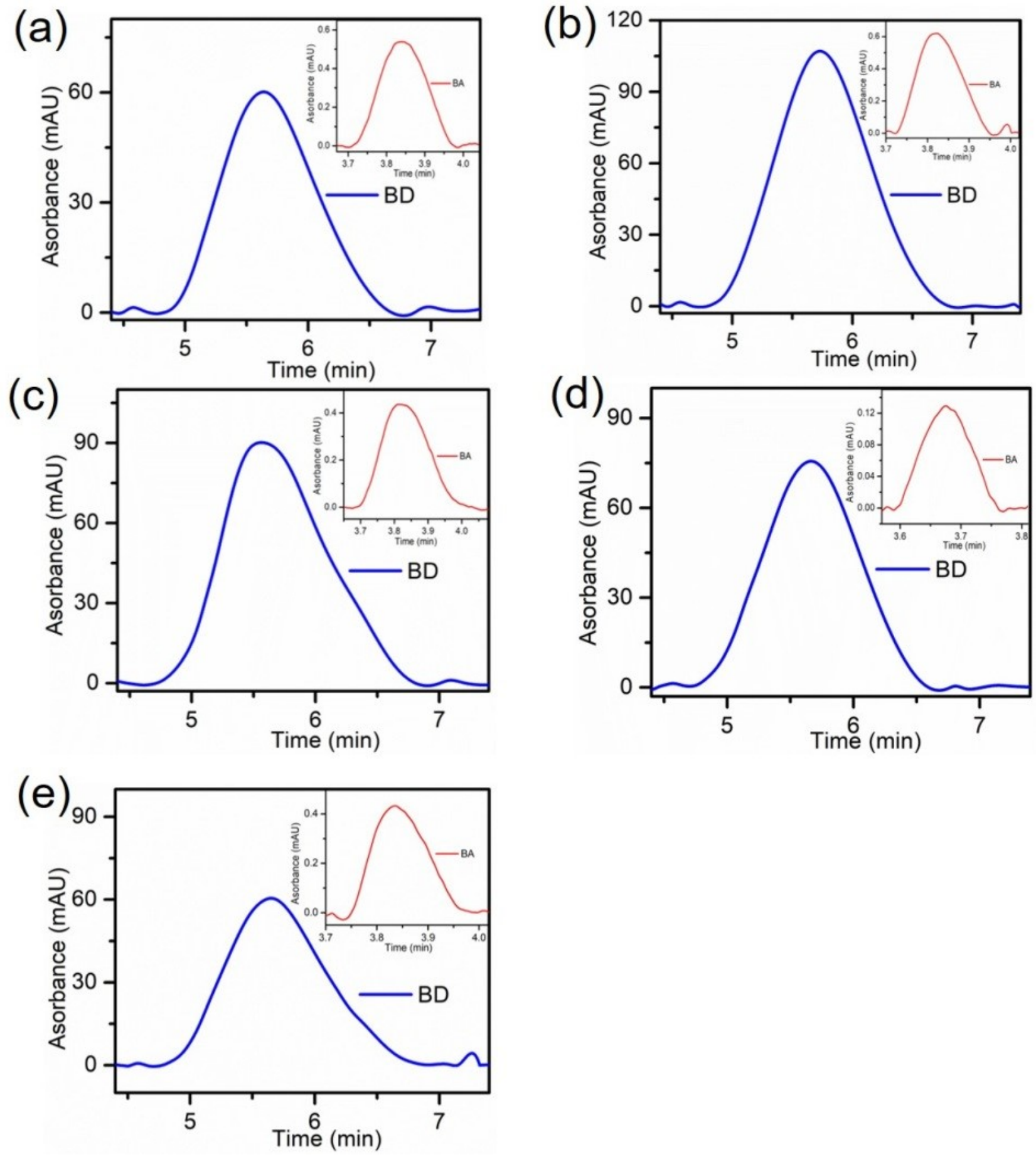

Figure S8. HPLC plots of photocatalytic toluene oxidation to produce BD and BA (in-set) in presence of oxygen by the different photocatalysts: (a) TiO_2-20 (b) TiO_2-30 (c) TiO_2-40 (d) TiO_2-50 and (e) P25 under UV light. Photocatalyst loading = 50 mg, solvent: acetonitrile and toluene (2:1), Light source= 24 Watt 360-nm UV light, volume = 30 ml, time = 20 hour.

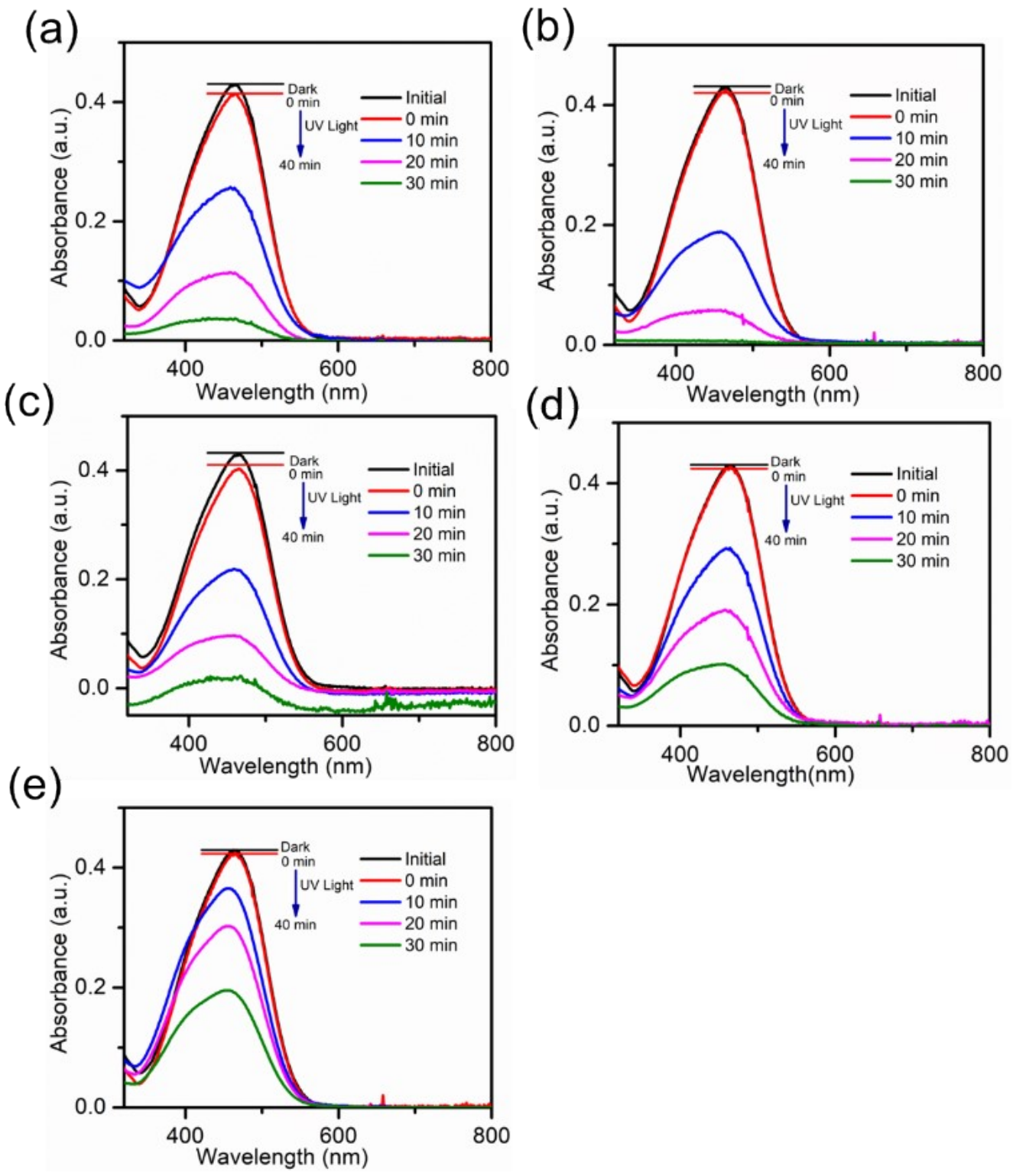

Figure S9. Photocatalytic degradation of MO dye by (a) TiO_2-20 (b) TiO_2-30 (c) TiO_2-40 (d) TiO_2-50 and (e) P25 under UV light. Photocatalyst loading = 20 mg, [MO] = 5×10^{-5} M, Light source= 24 Watt 360-nm UV light, volume = 50 ml.

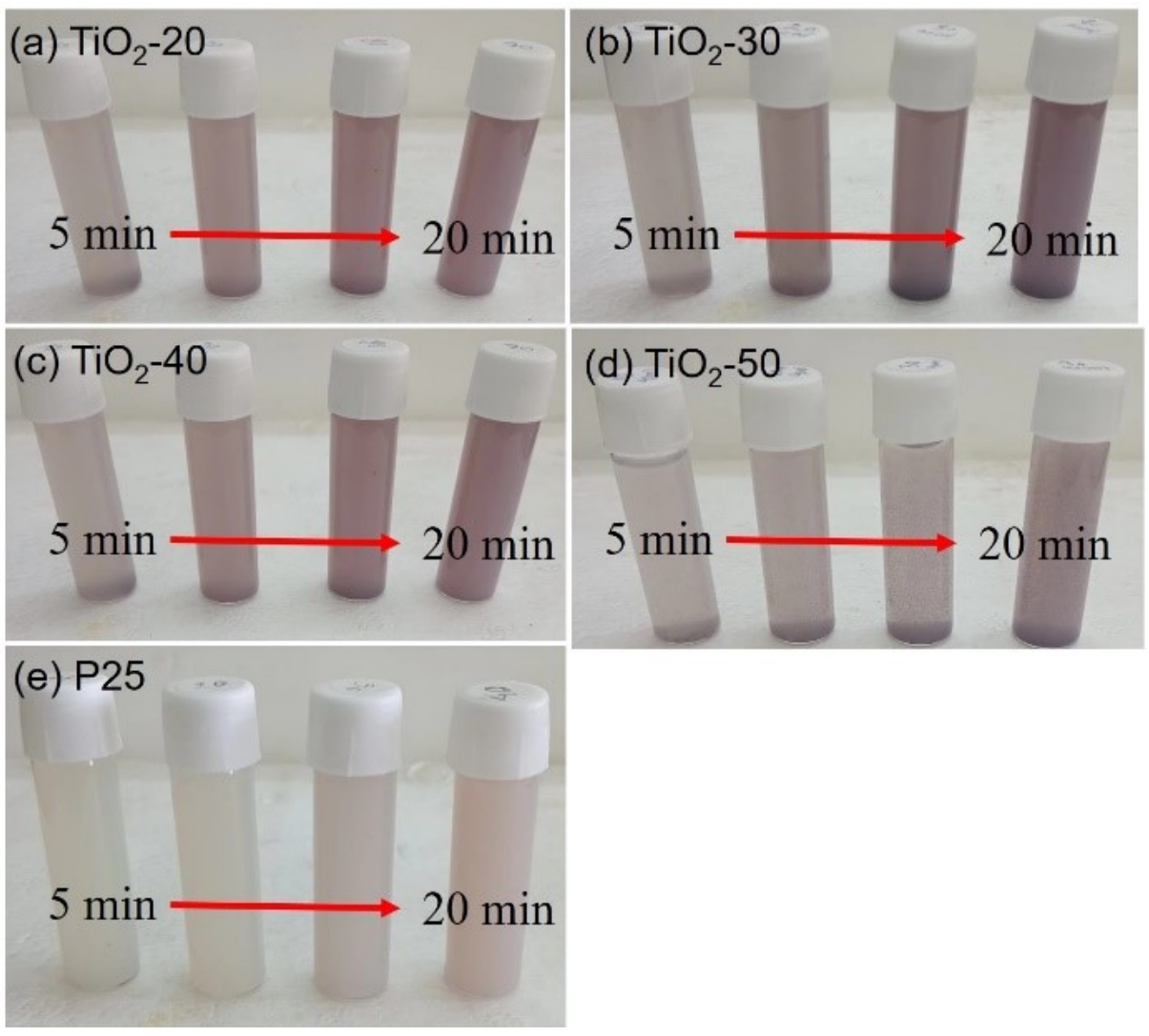

Figure S10. Photographs of photocatalytic conversion of NBT to purple coloured formazan in presence of oxygen by (a) TiO_2-20 (b)TiO_2-30 (c) TiO_2-40 (d) TiO_2-50 and (e) P25, respectively.

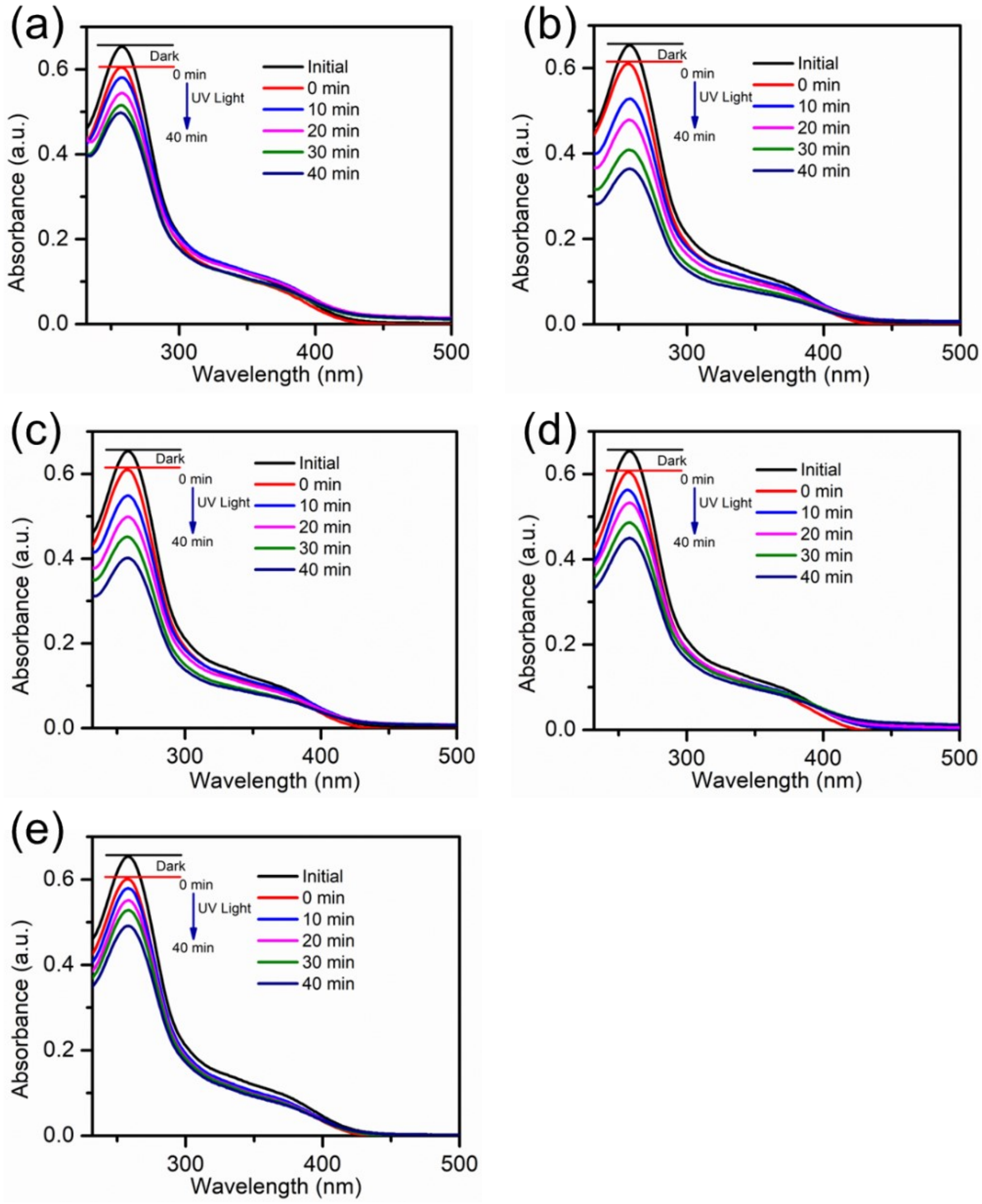

Figure S11. UV-Vis absorption spectra of nitroblue tetrazolium (NBT) reduction into formazan in presence of O_2 by (a) TiO_2-20 (b)TiO_2-30 (c) TiO_2-40 (d) TiO_2-50 and (e) P25 for 5 min time interval. Photocatalyst loading = 20 mg, [NBT] = 1.8×10^{-5} M, Light source= 24 Watt 360-nm UV light, volume = 25 ml.

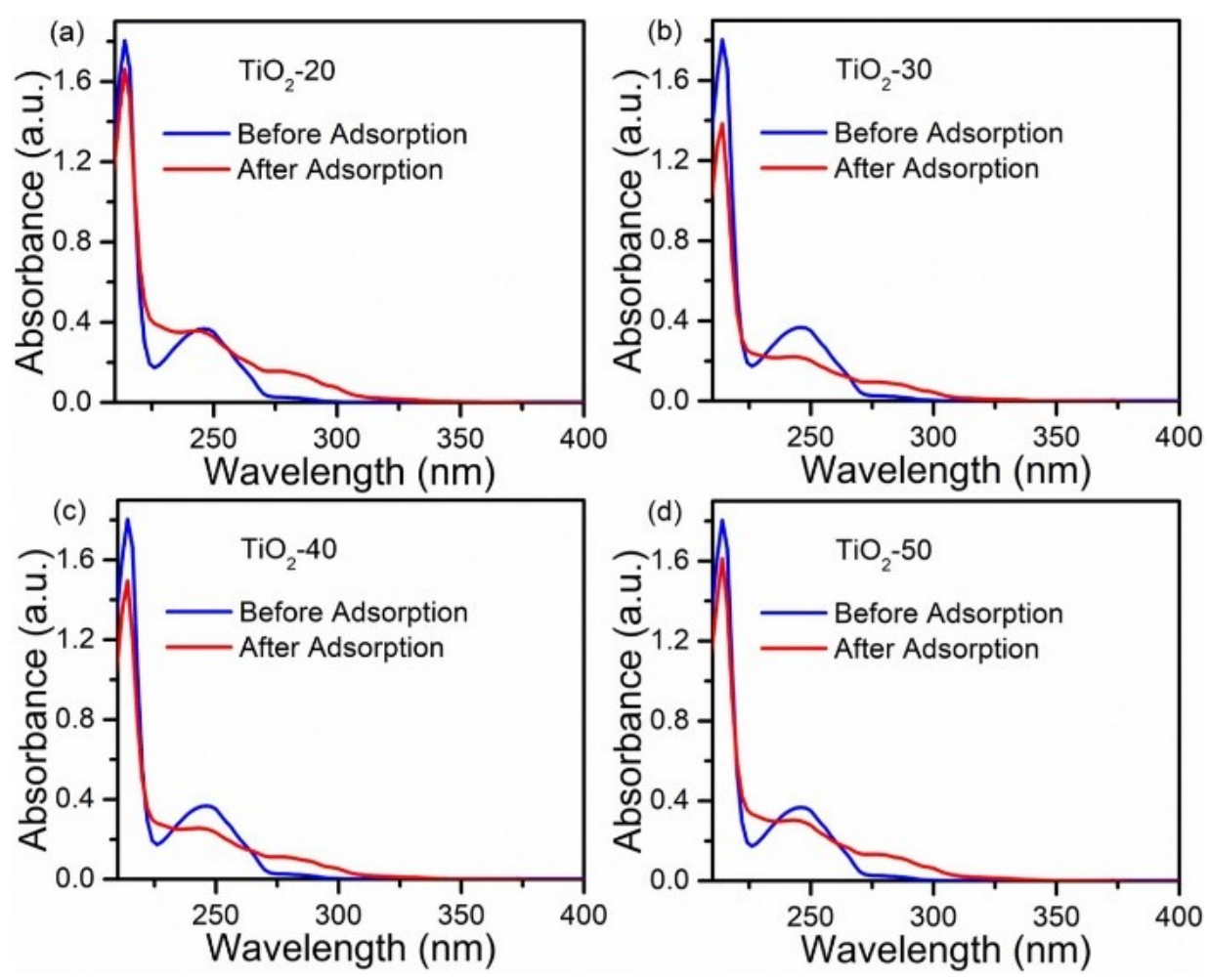

Figure S12. Adsorption studies of BA (100 ppm in acetonitrile) using different TiO_2 samples in dark.

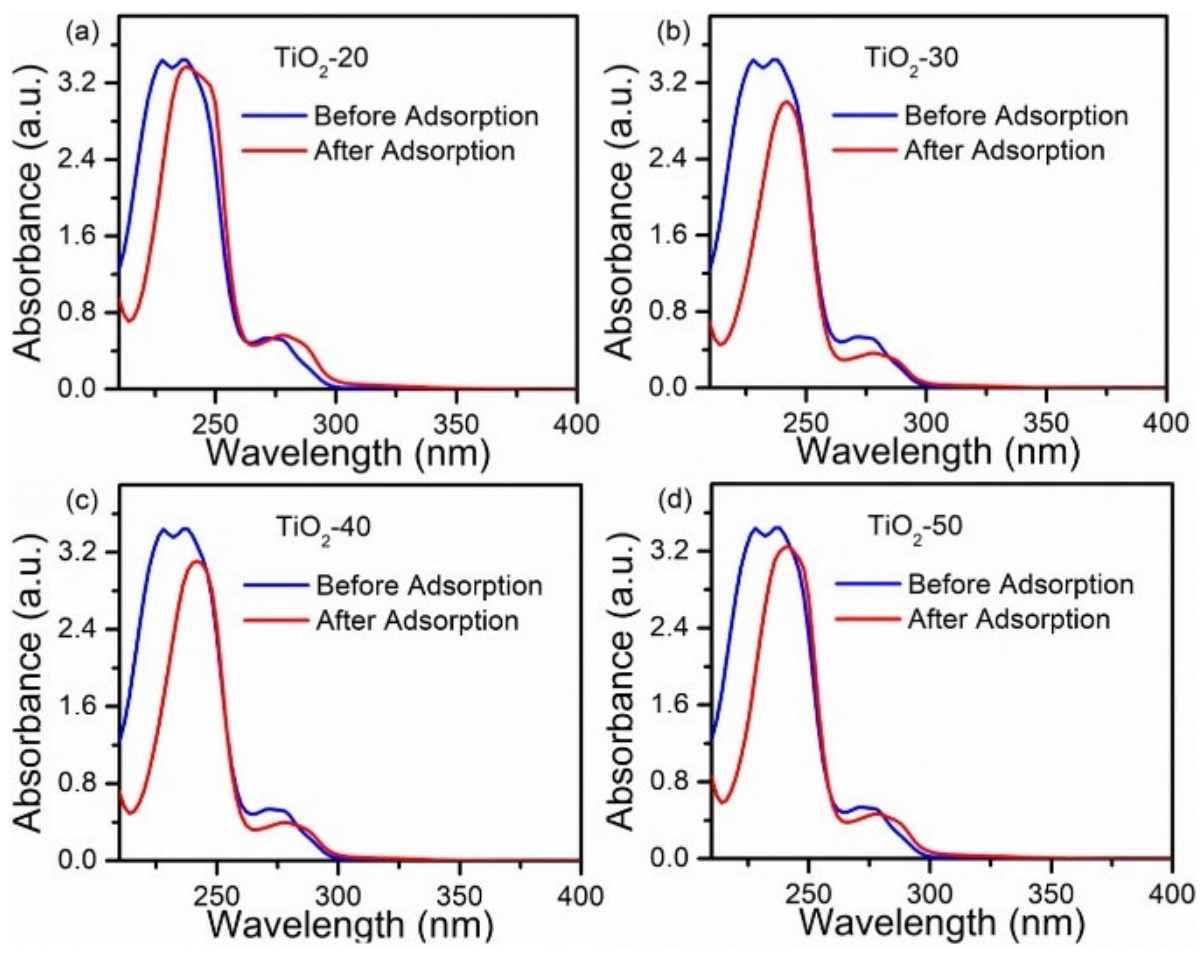

Figure S13. Adsorption studies of BD (50 ppm in acetonitrile) using different TiO_2 samples in dark.

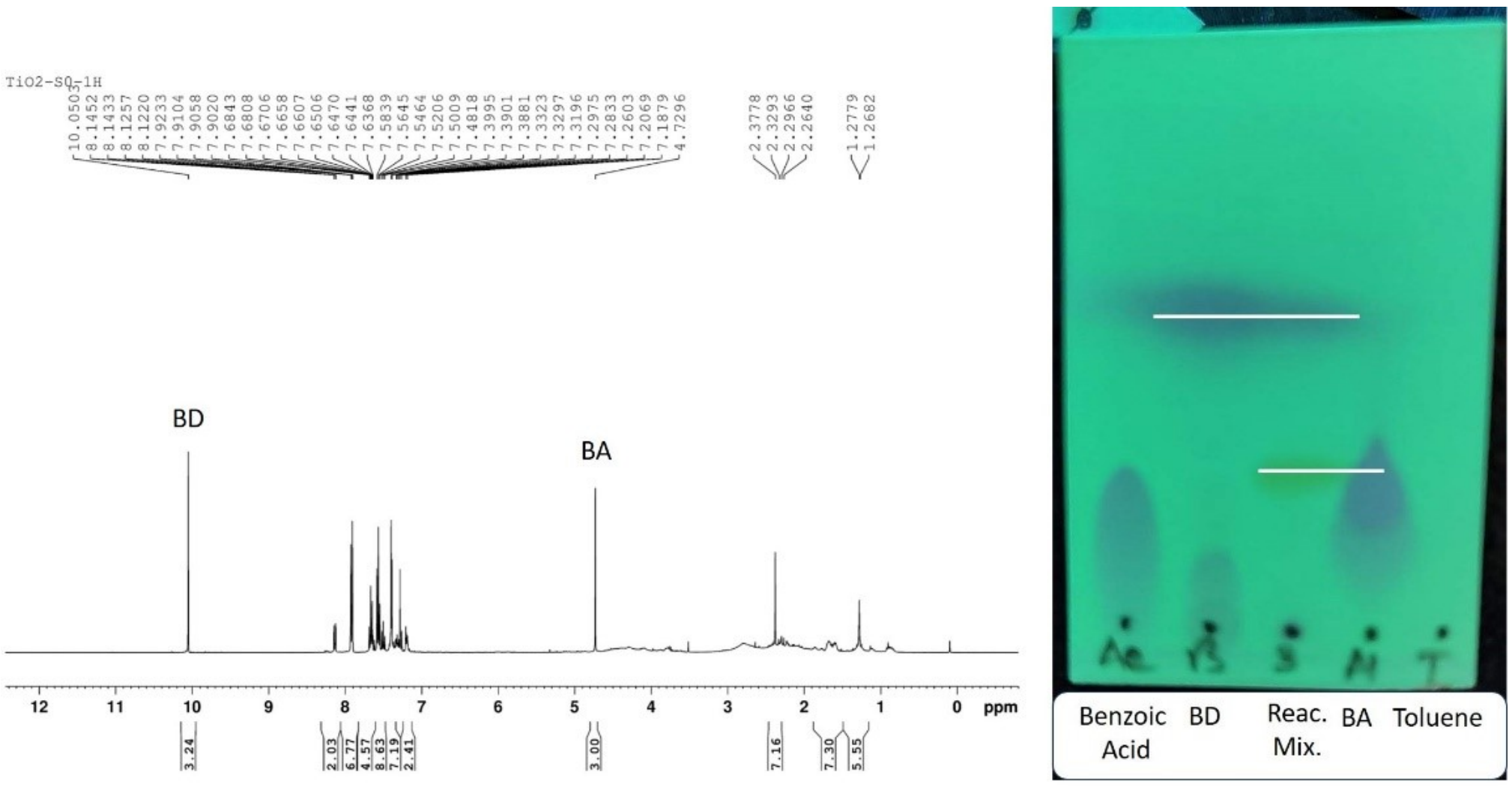

Figure S14. ^{1}H-NMR of the reaction mixture after photocatalytic reaction in $CDCl_3$ (left side) and photograph of TLC plate (right side).

Crystallite Size and Crystallinity Analysis:

The average crystallite size of the synthesized TiO_2 samples was estimated from the X-ray diffraction (XRD) patterns using the Scherrer equation:

$D = K\lambda/\beta cos\theta$

where, D is the crystallite size (nm), K is the Scherrer constant (0.9), λ is the wavelength of the radiation (0.15406 nm), β is the full width at half maximum (FWHM) (radian) , and θ is the peak position (radian).

The degree of crystallinity of the samples was estimated from the powder X-ray diffraction (XRD) patterns using an area-based method.

$$Crystallinity = \frac{Area\ of\ all\ Cryatalline\ peaks}{Area\ of\ all\ peaks\ (\ Crystalline\ + Amorphous)} \times 100$$

Table S3(a). Calculation of Average crystallite size for TiO_2-20.

2θ (°)	FWHM (°)	Crystallite Size (nm)	Average size (nm)
25.43	0.63	12.92699	11.36
38.02	0.76	11.05599	
48.14	0.73	11.91897	
54.13	0.58	15.38146	
55.07	1.01	8.870395	
62.99	1.16	8.03171	

Table S3(b). Calculation of crystallinity for TiO_2-20.

Area of crystalline peaks	Sum of Area of crystalline peaks	Area of all peaks	Crystallinity %
1293.6			
70.38			
359.21			
349.1	2875.33	5306.26	54.18
208.44			
195.36			
180.73			
52.56			
60.14			
105.77			

Table S4(a). Calculation of Average crystallite size for TiO_2-30.

2θ (°)	FWHM (°)	Crystallite Size (nm)	Average size (nm)
25.39	0.54	15.0803	12.59225
37.97	0.60	14.00215	
48.12	0.69	12.60894	
54.09	0.63	14.15818	
55.16	0.79	11.34528	
62.79	1.05	8.863663	
75.28	0.83	12.08723	

Table S4(b). Calculation of crystallinity for TiO_2-30.

Area of crystalline peaks	Sum of Area of crystalline peaks	Area of all peaks	Crystallinity %
896.93			
69.86			
195.42			
50.86	2276.97	3671.64	62.01
284.42			
180.81			
169.35			
170.32			
60.82			
66.38			
131.8			

Table S5(a). Calculation of Average crystallite size for TiO_2-40.

2θ (°)	FWHM (°)	Crystallite Size (nm)	Average size (nm)
25.39	0.37	22.00909	18.07028
37.93	0.35	24.00080	
48.14	0.49	17.75683	
54.06	0.48	18.58013	
55.17	0.55	16.29669	
62.83	0.60	15.51472	
68.95	0.55	17.52139	
70.41	0.60	16.20434	
75.23	0.68	14.74856	

Table S5(b). Calculation of crystallinity for TiO_2-40.

Area of crystalline peaks	Sum of Area of crystalline peaks	Area of all peaks	Crystallinity %
1745.06			
325.04			
482.71			
304.21	3770.91	6833.68	55.18
293.63			
270.03			
97.82			
102.13			
150.28			

Table S6(a). Calculation of Average crystallite size for TiO_2-50.

2θ ()	FWHM (°)	Crystallite Size (nm)	Average size (nm)
25.34	0.32	25.44551	26.83874
37.89	0.28	29.9974	
48.09	0.37	23.51122	
54.02	0.33	27.02084	
55.13	0.38	23.58302	
62.79	0.37	25.15364	
70.36	0.31	31.3536	
75.18	0.35	28.64472	

Table S6(b). Calculation of crystallinity for TiO_2-50.

Area of crystalline peaks	Sum of Area of crystalline peaks	Area of all peaks	Crystallinity %
1671.55			
122.96			
346.94			
117.6	3852.59	6909.83	55.75
450.94			
282.01			
245.55			
250.74			
86.07			
96.39			
143			
38.84			

Figure S15. Rod-like shape was calculated to be exposed with 1% {001}, 49% {100}, 33% {112} and 16% {101} facets whereas cube like exposed with 33.33% {100} and 66.66% {001} facets as per the crystallographic data (Length is 50 nm and width is 20 nm of the nanocrystals).

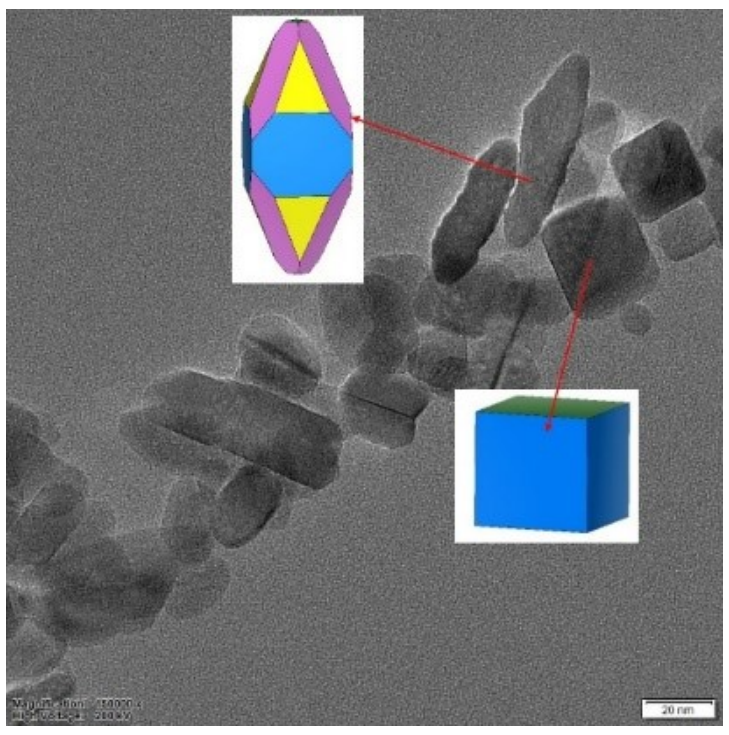

Supplement: Supplementary file 1 [file NA-008-D6NA00419A-s001.pdf]
